# Bioinformatics approach for structure modeling, vaccine design, and molecular docking of *Brucella* candidate proteins BvrR, OMP25, and OMP31

**DOI:** 10.1038/s41598-024-61991-7

**Published:** 2024-05-25

**Authors:** Alyaa Elrashedy, Mohamed Nayel, Akram Salama, Mohammed M. Salama, Mohamed E. Hasan

**Affiliations:** 1 Department of Animal Medicine and Infectious Diseases (Infectious Diseases), Faculty of Veterinary Medicine, University of Sadat City, Sadat City, Egypt; 2 Physics Department, Medical Biophysics Division, Faculty of Science, Helwan University, Cairo, Egypt; 3 Bioinformatics Department, Genetic Engineering and Biotechnology Research Institute, University of Sadat City, Sadat City, Egypt

**Keywords:** Brucellosis, Outer membrane protein, BvrR, Bioinformatics, Protein modeling, Epitopes prediction, Molecular docking, Protein structure predictions, Virtual drug screening

## Abstract

Brucellosis is a zoonotic disease with significant economic and healthcare costs. Despite the eradication efforts, the disease persists. Vaccines prevent disease in animals while antibiotics cure humans with limitations. This study aims to design vaccines and drugs for brucellosis in animals and humans, using protein modeling, epitope prediction, and molecular docking of the target proteins (BvrR, OMP25, and OMP31). Tertiary structure models of three target proteins were constructed and assessed using RMSD, TM-score, C-score, Z-score, and ERRAT. The best models selected from AlphaFold and I-TASSER due to their superior performance according to CASP 12 – CASP 15 were chosen for further analysis. The motif analysis of best models using MotifFinder revealed two, five, and five protein binding motifs, however, the Motif Scan identified seven, six, and eight Post-Translational Modification sites (PTMs) in the BvrR, OMP25, and OMP31 proteins, respectively. Dominant B cell epitopes were predicted at (44–63, 85–93, 126–137, 193–205, and 208–237), (26–46, 52–71, 98–114, 142–155, and 183–200), and (29–45, 58–82, 119–142, 177–198, and 222–251) for the three target proteins. Additionally, cytotoxic T lymphocyte epitopes were detected at (173–181, 189–197, and 202–210), (61–69, 91–99, 159–167, and 181–189), and (3–11, 24–32, 167–175, and 216–224), while T helper lymphocyte epitopes were displayed at (39–53, 57–65, 150–158, 163–171), (79–87, 95–108, 115–123, 128–142, and 189–197), and (39–47, 109–123, 216–224, and 245–253), for the respective target protein. Furthermore, structure-based virtual screening of the ZINC and DrugBank databases using the docking MOE program was followed by ADMET analysis. The best five compounds of the ZINC database revealed docking scores ranged from (− 16.8744 to − 15.1922), (− 16.0424 to − 14.1645), and (− 14.7566 to − 13.3222) for the BvrR, OMP25, and OMP31, respectively. These compounds had good ADMET parameters and no cytotoxicity, while DrugBank compounds didn't meet Lipinski's rule criteria. Therefore, the five selected compounds from the ZINC20 databases may fulfill the pharmacokinetics and could be considered lead molecules for potentially inhibiting *Brucella’s* proteins.

## Introduction

Brucellosis is considered one of the most hazardous infectious zoonotic diseases all over the world^1^. The disease is caused by *Brucella,* which is a Gram-negative, intracellular bacterial pathogen^2^. In contrast to other Gram-negative bacteria, *Brucella* has unique antigenic components that allow it to establish intracellular infection and induce virulence^3^. The BvrR/BvrS system, which is conserved among members of the *Alphaproteobacteria* class, is one of these virulence factors. It is the master regulator of gene expression required for host interaction, intracellular survival, expression of outer membrane proteins (OMPs), and lipopolysaccharides (LPS) structure. It also controls the expression of the virB operon, which encodes for the type IV secretion system (T4SS), responsible for secreting bacterial factors necessary for modulating the maturation of the *Brucella*-containing vacuoles (BCVs)^4^. In addition, OMP25, a *Brucella* outer membrane protein that triggers a potent immunological response, is highly conserved among different types and subtypes of Brucellae^5^. OMP25 protein has a role in macrophages thriving intracellularly in the TNF-α production reduced and IL-12-negative regulation at both the transcriptional and post-transcriptional levels achieved^6^. It plays multiple roles which are inhibition of interferon β (INF-β) and activation of IFN-stimulated genes (ISGs)^7^.

Moreover, OMP31 is a key component of *Brucella's* most important virulence factors and is essential for the defense against *Brucella* infection^8^. It plays a role in mediating attachment, entry, survival within host cells, and avoiding immune cell detection and suppressing their function. In addition, it interferes with dendritic cell maturation and function, and induces programmed cell death of infected macrophages, thereby helping *Brucella* to persist in the host^9^. Moreover, it has high antigenicity which allows it to be more candidate for peptide vaccine development^10^. Therefore, the identification of these virulence genes that encode different important proteins is crucial for vaccine design in controlling brucellosis.

Bioinformatics plays important roles not only in handling large volumes of data, but also in predicting, analyzing, or interpreting clinical and preclinical findings, drug discovery, drug assessment, and drug development^11^. Protein structure prediction approaches have emerged as important proteomic analysis for understanding phenomena in modern molecular and cell biology, and have significant applications in biotechnology and medicine^12^. These approaches revolve around predicting the three-dimensional (3-D) structure of a protein from its primary structure (amino acid sequence) and are divided into three groups: Comparative modeling (also known as Homology modeling), Fold recognition (also known as Threading), and *Ab-initio* prediction (also known as Free modeling). The homology modeling technique tries to predict the unknown structure based on a template (a known structure), whose similarity to the sequence is higher than 35% ^13^. On the other hand, for domains that have no homology, fold recognition uses a library of templates, or *ab-initio* techniques are used. *Ab-initio* approaches can be used to predict the protein structure from the sequence data when suitable structure templates are unavailable^14^.

There are considerable knowledge deficits in creating new vaccines or drug molecules. Bioinformatics capabilities can help overcome these shortcomings of the live attenuated *Brucella’s* vaccine and develop a safe and powerful subunit vaccine^15^. Another one of the most important applications in bioinformatics is epitope prediction, which predicts a variety of specific epitopes for B cell recognition and T cell through MHC class I and II molecules^16^. Various techniques and resources are utilized in epitope prediction, encompassing sequence-based methodologies (like ABCpred), structural approaches (such as DiscoTope), immunoinformatics-based strategies (involving NetMHC, NetMHCII, and SYFPEITHI), and machine learning-based models (likewise incorporating SVM)^17^. This approach is cost and time-efficient compared to laboratory testing and allows the selection of immunogenic regions from the pathogen genomes^5^.

In this study, we plan to utilize multi-techniques like protein structure prediction, epitope prediction and vaccine design, molecular docking, and ADMET prediction. The main emphasis is used for three protein targets (BvrR, OMP25, and OMP31) as potential vaccine candidates and medicines for the development of new human and animal brucellosis drugs and vaccines. Through the utilization of these advanced tools and techniques, the research aims to develop next-generation drugs and vaccines for this disease.

## Results

### Domain separation

Domain separation of the three proteins (BvrR, OMP25, and OMP31) is carried out through CDD and SMART servers. CDD showed that BvrR, OMP25, and OMP31 proteins had only one domain (10–236, 5–213, and 9–261) with E-value (1.27e–81, 1.44e–42, and 1.91e–36) for the three proteins, respectively (Table 1). SMART displayed two, three, and five domains for BvrR, OMP25, and OMP31 proteins, respectively as listed in (Table 2).

**Table 1:** Domain assessment of three target proteins using CDD server.

Protein	Name	Accession	Description	Interval	E-value
BvrR	OmpR	COG0745	DNA-binding response regulator, the OmpR family, contains REC and winged-helix (wHTH) domain	10–236	1.27e–81
OMP25	LomR	COG3637	Opacity protein and related surface antigens [membrane/envelope biosynthesis/Cell wall]	5–213	1.44e–42
OMP31				9–261	1.91e–36

**Table 2:** Domain assessment of three target proteins using SMART server.

Proteins	Name	Accession	Description	Interval	E-value	Gene ontology (GO)
BvrR	REC	SM000448	cheY-homologous receiver domain regulates the clockwise rotation of *E. coli* flagellar motors. This domain contains a phosphoacceptor site that is phosphorylated by histidine kinase homologues	8–118	2.03e–38	Phosphorelay signal transduction system (GO:0000160)
	Trans_reg_C	SM000862	Transcriptional regulatory protein, C terminal. This domain is almost always associated with the response regulator receiver domain. It may play a role in DNA binding	158–234	3.54e–20	Phosphorelay signal transduction system (GO:0000160), regulation of transcription, DNA-templated (GO:0006355), DNA binding (GO:0003677)
OMP25	OMP_b-brl	PF13505	This domain is found in a wide range of outer membrane proteins. This domain assumes a membrane-bound beta-barrel fold	9–213	2.3e–26	–
	OmpA_membrane	PF01389	–	46–213	2.6e–9	Cell outer membrane (GO:0009279), an integral component of the membrane (GO:0016021)
	Opacity	PF02462	–	78–213	0.0000012	Membrane (GO:0016020), porin activity (GO:0015288)
OMP31	OMP_b-brl	PF13505	This domain is found in a wide range of outer membrane proteins. This domain assumes a membrane-bound beta-barrel fold	13–261	2.5e–26	–
	Autotransporter	PF03797	–	40–222	4.2e–07	–
	OmpA_membrane	PF01389	–	45–261	5.4e–11	Integral component of membrane (GO:0016021), cell outer membrane (GO:0009279)
	Opacity	PF02462	–	115–261	7.8e–09	Membrane (GO:0016020), porin activity (GO:0015288)

### Secondary structure prediction

To increase the accuracy of secondary structure prediction, the result was obtained from nine neural network prediction servers: PSIPRED, NPS@ SOPMA, CFSSP, PSSpred, Lambada Predict Protein, GOR, PredictProtein, PROTEUS2, and RaptorX secondary structure prediction for the three target proteins. The predictprotein was the best and most accurate server for secondary structure prediction, composed of alpha helix 36.29%, 11.74%, and 8.05%; beta sheet 22.36%, 43.19%, and 38.70%; random coil 41.35%, 45.07% and 53.26%; exposed 59.92%, 46.48% and 45.98% and buried 40.08%, 53.52% and 54.02% for BvrR, OMP25 and, OMP31 proteins, respectively (Table 3).

**Table 3:** Secondary structure and solvent accessibility prediction.

Server	protein	Helix (H)	Sheet (E)	Turn (T)/coil (C)	Exposed (e)	Buried (b)
CFSSP	BvrR	82.7%	59.1%	14.3%	–	–
	OMP25	62.4%	65.3%	13.6%	–	–
	OMP31	65.5%	68.6%	15.7%	–	–
PredictProtein	**BvrR**	**36.29%**	**22.36%**	**41.35%**	**59.92%**	**40.08%**
	**OMP25**	**11.74%**	**43.19%**	**45.07%**	**46.48%**	**53.52%**
	**OMP31**	**8.05%**	**38.70%**	**53.26%**	**45.98%**	**54.02%**
SOPMA	BvrR	32.91%	20.68%	46.41%	–	–
	OMP25	22.07%	22.07%	55.87%	–	–
	OMP31	17.62%	18.77%	63.60%	–	–

Significant values are in **bold**.

### 3-D protein structure

To construct the 3-D model, initial models were built, refined, and evaluated, and finally, the model with the highest quality was chosen (Tables 4, 5 and 6).

**Table 4:** Assessment of predicted 3-D Structure of BvrR protein using different servers according to CASP15.

		RMSD	TM-score	Z-score	QMEAN	ERRAT	MolProbity score	Clash score	Ramachandran favored
DeepRefiner	Alphafold colab	1.30	0.769	16.943	0.74	92.8889	2.82	183.84	97.55%
	CEthreader	1.49	0.731	17.069	0.75	99.5614	2.75	194.88	98.16%
	C-QUARK	1.17	0.771	16.940	0.79	98.6784	3.29	184.73	89.57%
	I-TASSER	1.75	0.9212	16.876	0.70	97.8166	2.81	180.75	97.55%
	LOMETS	1.36	0.769	17.024	0.73	98.2456	2.98	190.81	96.34%
	Phyre2	3.46	0.906	31.161	0.58	57.4324	2.93	152.71	95.87%
	Robetta	1.05	0.733	17.115	0.74	96.5066	2.71	178.77	100.00%
	SWISS-MODEL	1.13	0.765	16.894	0.73	95.5357	2.91	184.57	96.89%
ModRefiner	Alphafold colab	1.20	0.9953	16.825	0.74	90.583	2.10	42.18	99.15%
	CEthreader	1.50	0.9872	16.826	0.70	92.5439	2.57	56.85	96.60%
	C-QUARK	1.11	0.9939	16.935	0.70	82.9694	3.04	64.19	90.21%
	I-TASSER	0.61	0.9896	17.045	0.71	85.1528	2.54	58.95	95.74%
	LOMETS	1.34	0.90	16.824	0.71	79.9107	2.43	60.70	97.83%
	Phyre2	3.14	0.9852	31.318	0.61	71.3178	2.34	36.49	95.67%
	Robetta	1.06	0.9932	17.015	0.74	89.9563	2.07	38.77	98.72%
	SWISS-MODEL	1.17	0.9885	16.716	0.73	78.5714	2.26	60.96	98.26%
GalaxyWeb	Alphafold colab	1.13	0.781	17.083	0.75	95.9641	1.20	4.19	99.15%
	CEthreader	1.46	0.774	17.019	0.73	93.8053	1.57	7.33	97.02%
	C-QUARK	1.08	0.735	17.008	0.71	87.3362	2.37	11.00	91.06%
	I-TASSER	0.38	0.9957	17.139	0.73	96.9298	1.73	8.64	96.17%
	LOMETS	1.01	0.817	16.781	0.74	95.9276	1.55	6.02	96.60%
	phyre2	3.32	0.908	31.221	0.60	82.4742	1.39	2.26	94.23%
	Robetta	1.00	0.756	16.646	0.75	99.095	1.20	4.19	98.72%
	SWISS-MODEL	1.00	0.769	16.996	0.76	91.3636	1.71	9.09	98.26%
trRosetta	Alphafold colab	3.03	0.897	27.896	0.62	71.5	1.29	3.49	97.16%
	CEthreader	1.26	0.780	17.067	0.76	97.7679	0.84	1.09	97.87%
	C-QUARK	1.23	0.725	16.897	0.77	93.2432	0.69	0.54	99.15%
	I-TASSER	1.05	0.775	17.118	0.76	98.2063	0.98	1.36	97.45%
	LOMETS	1.32	0.818	16.755	0.77	96	0.81	1.09	98.72%
	phyre2	3.40	0.902	31.154	0.69	79.6875	1.16	3.68	98.08%
	Robetta	1.27	0.773	16.866	0.77	96.4286	0.87	1.36	98.30%
	SWISS-MODEL	2.22	0.783	16.940	0.78	99.0868	0.60	0.28	98.70%
PREFMD	Alphafold colab	1.59	0.813	16.945	0.72	96	0.86	0.26	96.17%
	CEthreader	1.25	0.776	16.972	0.72	98.6842	0.76	0.00	96.17%
	C-QUARK	1.23	0.791	17.015	0.70	95.4955	1.07	0.26	92.34%
	I-TASSER	1.68	0.9224	16.863	0.71	90.2222	0.67	0.00	97.02%
	LOMETS	–	–	–	–	–	–	–	–
	phyre2	3.60	0.863	31.035	0.60	68.7117	1.47	1.29	87.02%
	Robetta	1.30	0.820	16.931	0.73	93.7778	0.53	0.00	97.87%
	SWISS-MODEL	1.39	0.804	16.843	0.74	93.578	0.50	0.00	98.26%
ReFOLD	Alphafold colab	1.21	0.797	16.832	0.75	96.9432	1.65	9.44	97.87%
	CEthreader	1.55	0.795	16.860	0.71	97.3568	2.86	18.34	94.47%
	C-QUARK	1.10	0.816	16.860	0.71	96.5066	2.70	9.43	91.49%
	I-TASSER	0.72	0.9847	16.844	0.72	93.0131	2.98	20.96	94.89%
	LOMETS	0.97	0.801	17.099	0.73	96.9432	2.11	10.22	97.02%
	Phyre2	–	–	–	–	–	–	–	–
	Robetta	1.10	0.786	16.888	0.75	90.625	1.73	3.40	97.45%
	SWISS-MODEL	1.01	0.781	16.943	0.74	95.5357	2.56	13.37	95.22%

**Table 5:** Assessment of predicted 3-D structure of OMP25 protein using different servers according to CASP15.

		RMSD	TM-score	Z-score	QMEAN	ERRAT	MolProbity score	Clash score	Ramachandran favored
DeepRefiner	Alphafold colab	1.94	0.888	27.890	0.55	53.2967	3.18	156.92	90.98%
	CEthreader	2.50	0.883	27.893	0.52	81.2183	3.09	168.15	93.98%
	C-QUARK	2.75	0.883	27.895	0.45	76.0976	3.37	171.28	84.21%
	I-TASSER	0.84	0.9765	27.889	0.48	80.1105	3.71	193.99	77.44%
	LOMETS	2.17	0.5969	28.073	0.48	90.8163	3.28	153.97	86.36%
	Phyre2	3.81	0.960	20.836	0.90	71.5278	2.64	151.41	98.10%
	Robetta	2.03	0.880	27.887	0.65	58.4158	2.69	169.97	98.50%
	SWISS-MODEL	2.34	0.981	22.555	0.62	77.0833	3.04	166.78	94.79%
ModRefiner	Alphafold colab	1.95	0.9569	27.904	0.52	73.6264	2.33	32.64	95.26%
	CEthreader	2.78	0.9810	27.895	0.51	92.5439	2.54	46.18	95.26%
	C-QUARK	2.91	0.9832	27.895	0.40	65.8537	3.20	59.73	85.31%
	I-TASSER	0.94	0.9760	27.891	0.49	57.6087	3.21	67.12	87.20%
	LOMETS	2.06	0.9727	27.895	0.53	76.4103	2.40	33.87	94.31%
	Phyre2	3.27	0.9899	20.828	0.91	72.2222	2.32	30.11	94.77%
	Robetta	2.02	0.9917	27.883	0.62	65.1515	2.27	32.34	96.68%
	SWISS-MODEL	2.21	0.9882	22.558	0.59	64.7436	2.19	26.16	95.83%
GalaxyWeb	Alphafold colab	1.94	0.880	27.892	0.54	86.5169	1.16	3.69	99.05%
	CEthreader	2.66	0.893	27.884	0.49	68.1564	1.82	8.62	94.79%
	C-QUARK	2.90	0.882	27.888	0.43	63.4146	2.24	12.31	86.26%
	I-TASSER	0.75	0.9892	27.892	0.48	80.791	2.35	15.39	84.83%
	LOMETS	2.02	0.882	27.887	0.49	79.7814	1.84	3.69	87.20%
	phyre2	3.06	0.917	30.979	0.61	85.1485	1.46	2.58	93.75%
	Robetta	2.01	0.889	27.895	0.63	78.0612	1.21	4.31	98.58%
	SWISS-MODEL	2.21	0.982	22.552	0.61	89.1156	1.29	1.92	95.24%
trRosetta	Alphafold colab	3.29	0.885	27.699	0.63	78.3898	1.08	2.90	98.07%
	CEthreader	1.97	0.880	27.893	0.60	71.8593	1.25	2.22	96.21%
	C-QUARK	1.98	0.891	27.892	0.61	64.5833	1.12	2.54	97.63%
	I-TASSER	1.96	0.879	27.893	0.62	64.433	1.04	2.54	98.10%
	LOMETS	2.01	0.892	27.887	0.62	64.5503	1.19	2.54	97.16%
	phyre2	3.42	0.930	30.965	0.68	73.8462	1.13	2.01	97.12%
	Robetta	1.98	0.892	27.894	0.62	63.4921	1.08	2.22	97.63%
	SWISS-MODEL	3.15	0.985	22.556	0.72	69.1824	1.15	2.79	97.62%
PREFMD	Alphafold colab	2.08	0.878	27.893	0.55	67.3575	1.06	0.00	89.57%
	CEthreader	2.61	0.879	27.892	0.48	78.4946	0.95	0.00	92.89%
	C-QUARK	2.89	0.895	27.892	0.44	68.5	1.11	0.00	87.68%
	I-TASSER	3.47	0.914	27.887	0.53	57.513	1.12	0.00	87.20%
	LOMETS	2.06	0.880	27.899	0.51	59.7938	1.24	0,62	90.52%
	phyre2	3.19	0.954	20.823	0.86	53.2374	0.66	0.00	98.04%
	Robetta	3.07	0.885	27.895	0.63	66.1765	0.65	0.00	97.16%
	SWISS-MODEL	3.01	0.976	22.552	0.63	66.4474	0.95	0.00	92.86%
ReFOLD	Alphafold colab	3.39	0.894	27.892	0.56	62.1053	2.36	14.17	91.47%
	CEthreader	2.62	0.887	27.892	0.49	65.3266	2.54	11.70	95.73%
	C-QUARK	3.65	0.888	27.892	0.44	88.2927	3.34	30.51	83.41%
	I-TASSER	2.97	0.880	27.892	0.53	74.1294	3.16	22.48	85.31%
	LOMETS	2.06	0.6024	27.894	0.52	69.2308	2.86	11.70	88.15%
	Phyre2	–	–	–	–	–	–	–	–
	Robetta	3.46	0.889	27.900	0.65	73.1707	2.45	18.48	96.68%
	SWISS-MODEL	3.59	0.985	22.553	0.61	80.6452	2.56	13.08	93.45%

**Table 6:** Assessment of predicted 3-D Structure of OMP31 protein using different servers according to CASP15.

		RMSD	TM-score	Z-score	QMEAN	ERRAT	MolProbity score	Clash score	Ramachandran favored
DeepRefiner	Alphafold colab	2.42	0.898	27.691	0.55	75.4717	2.84	139.11	96.41%
	CEthreader	2.77	0.894	27.694	0.55	66.9323	2.94	174.00	96.41%
	C-QUARK	3.03	0.895	27.688	0.36	53.7849	3.40	171.33	82.04%
	I-TASSER	2.68	0.876	27.699	0.34	77.6699	3.46	176.89	78.44%
	LOMETS	–	–	–	–	–	–	–	–
	Phyre2	2.49	0.892	27.894	0.65	57.7689	2.94	152.89	95.81%
	Robetta	2.76	0.982	21.731	0.52	80.814	2.91	134.49	95.45%
	SWISS-MODEL	2.45	0.9488	27.686	0.50	78.744	1.88	24.62	98.07%
ModRefiner	Alphafold colab	2.51	0.9834	27.699	0.51	56.5957	2.91	70.74	92.66%
	CEthreader	2.95	0.9850	27.694	0.41	49.2063	3.01	62.97	79.54%
	C-QUARK	1.11	0.9065	27.693	0.37	59.1398	3.18	64.01	84.17%
	I-TASSER	3.87	0.9778	17.601	0.86	88.6792	2.36	39.33	95.89%
	LOMETS	–	–	–	–	–	–	–	–
	Phyre2	2.95	0.9898	21.740	0.50	71.4286	2.38	38.13	95.37%
	Robetta	2.42	0.906	27.692	0.51	82.3834	1.24	4.66	99.61%
	SWISS-MODEL	2.55	0.875	27.691	0.50	58.296	2.29	12.69	93.05%
GalaxyWeb	Alphafold colab	2.92	0.890	27.700	0.40	52.6316	2.50	13.21	79.92%
	CEthreader	0.66	0.9881	27.694	0.37	47.9532	2.99	41.19	77.99%
	C-QUARK	3.21	0.916	31.016	0.61	87.6289	1.26	1.94	95.67%
	I-TASSER	2.39	0.884	27.689	0.62	74.4589	1.33	4.92	97.68%
	LOMETS	–	–	–	–	–	–	–	–
	phyre2	2.37	0.894	27.696	0.63	83.0579	1.28	2.64	96.53%
	Robetta	2.38	0.900	27.691	0.64	81.07	1.25	2.11	96.14%
	SWISS-MODEL	2.41	0.888	27.689	0.64	80.1619	1.23	2.64	96.91%
trRosetta	Alphafold colab	2.38	0.893	27.699	0.65	81.8182	1.05	2.11	97.68%
	CEthreader	3.26	0.880	17.458	0.86	94.8357	1.00	1.78	97.72%
	C-QUARK	2.35	0.894	27.692	0.64	76.4463	1.02	2.38	98.07%
	I-TASSER	2.77	0.956	21.756	0.68	71.9388	1.04	2.53	99.54%
	LOMETS	–	–	–	–	–	–	–	–
	phyre2	2.81	0.895	27.695	0.52	50.2146	1.11	0.26	91.12%
	Robetta	3.15	0.888	27.692	0.41	70	1.15	0.00	85.33%
	SWISS-MODEL	3.04	0.892	27.696	0.36	72	1.50	0.00	82.24%
PREFMD	Alphafold colab	3.91	0.857	17.517	0.86	97.6526	0.75	0.00	97.65%
	CEthreader	2.42	0.903	27.692	0.60	73.5931	0.82	0.00	95.37%
	C-QUARK	2.66	0.949	21.734	0.50	72.2826	0.93	0.00	93.52%
	I-TASSER	2.52	0.910	27.700	0.53	61.9469	2.40	9.84	93.44%
	LOMETS	–	–	–	–	–	–	–	–
	phyre2	2.90	0.900	27.700	0.40	67.9842	3.15	19.69	81.08%
	Robetta	1.59	0.9403	27.684	0.36	75.9563	3.24	16.84	73.75%
	SWISS-MODEL	2.68	0.876	27.699	0.34	77.6699	3.46	176.89	78.44%
ReFOLD	Alphafold colab	2.53	0.871	27.688	0.64	68.3794	2.59	16.08	95.75%
	CEthreader	2.73	0.934	21.755	0.51	73.2759	1.82	1.24	92.59%
	C-QUARK	2.42	0.898	27.691	0.55	75.4717	2.84	139.11	96.41%
	I-TASSER	2.77	0.894	27.694	0.55	66.9323	2.94	174.00	96.41%
	LOMETS	–	–	–	–	–	–	–	–
	Phyre2	–	–	–	–	–	–	–	–
	Robetta	3.84	0.842	17.514	0.88	95.7143	2.65	154.17	98.60%
	SWISS-MODEL	2.49	0.892	27.894	0.65	57.7689	2.94	152.89	95.81%

#### Construction of initial model using target-template alignment

While Swiss-Model, AlphaFold, CEthreader, and LOMETS were intended for the aligned regions, I-TASSER, C-QUARK, Robetta, and Phyre2 were designed for low-similarity regions to construct the structural models for the unaligned regions. I-TASSER developed the top five models based on the confidence score (C-score) of each model, which was equal to -0.18 for BvrR protein, − 2.14 for OMP25 protein, and − 3.19 for OMP31 protein which was typically in the range of (− 5, 2). The TM-score (0.36 ± 0.12) and RMSD (13.5 ± 4.0 Å) were estimated based on the C-score and protein length. C-QUARK server constructed the best ten correct protein 3-D models from amino acid sequences based on estimated TM-score (0.634 ± 0.006, 0.563 ± 0.027, and 0.494 ± 0.076) for BvrR, OMP25, and OMP31 proteins, respectively. Each query sequence was given five models by GalaxyWEB, which also chose templates for modeling by rescoring HHsearch results. While Phyre2 built 3-D models using sophisticated distant homology detection techniques, SWISS-MODEL provided ten models for BvrR protein, eight models for OMP25 protein, and six models for OMP31 protein based on QMEAN score (0.74, 0.59, and 0.49), respectively. LOMETS created the top 10 models based on the C-score (medium) and Z-score (7.10, 1.98) of the template for BvrR and OMP25 proteins, respectively. Also, based on the Z-score (2.16) (normalized Z-score ≥ 1 means a good alignment) for the three proteins, CEthreader created the top 20 templates.

#### Reduced-level structure assembly and refinement simulations

The second stage of structure prediction involved the refining of the three proteins (BvrR, OMP25, and OMP31). In terms of hydrogen bonds, backbone structure, and side-chain positioning, the results from the DeepRefiner, Modrefiner, Galaxy WEB, trRosetta, PREFMD, and ReFOLD servers succeed in bringing the basic starting models closer to their native state. Our results from the I-TASSER server revealed improved topology of the predicted 3-D structure by lowering the RMSD score (0.38, 0.75, and 0.661), the clash score (8.64, 15.39, and 41.19) and increasing the TM-score (0.9997, 0.9892 and 0.9881), for BvrR, OMP25 and, OMP31 proteins, respectively. In addition, the refinement process of the initial model from AlphaFold decreased the RMSD score (1.13, 1.94, and 2.42), the clash score (4.19, 3.69, and 4.66) and increased the TM-score (0.781, 0.880 and 0.906), for BvrR, OMP25 and OMP31 proteins, respectively. It also provided a significant enhancement in the physical quality of local and global structures compared to the initial model produced by a chosen server, such as the I-TASSER and AlphaFold servers for the target proteins.

#### Model evaluation and selection

**Figure 1:**
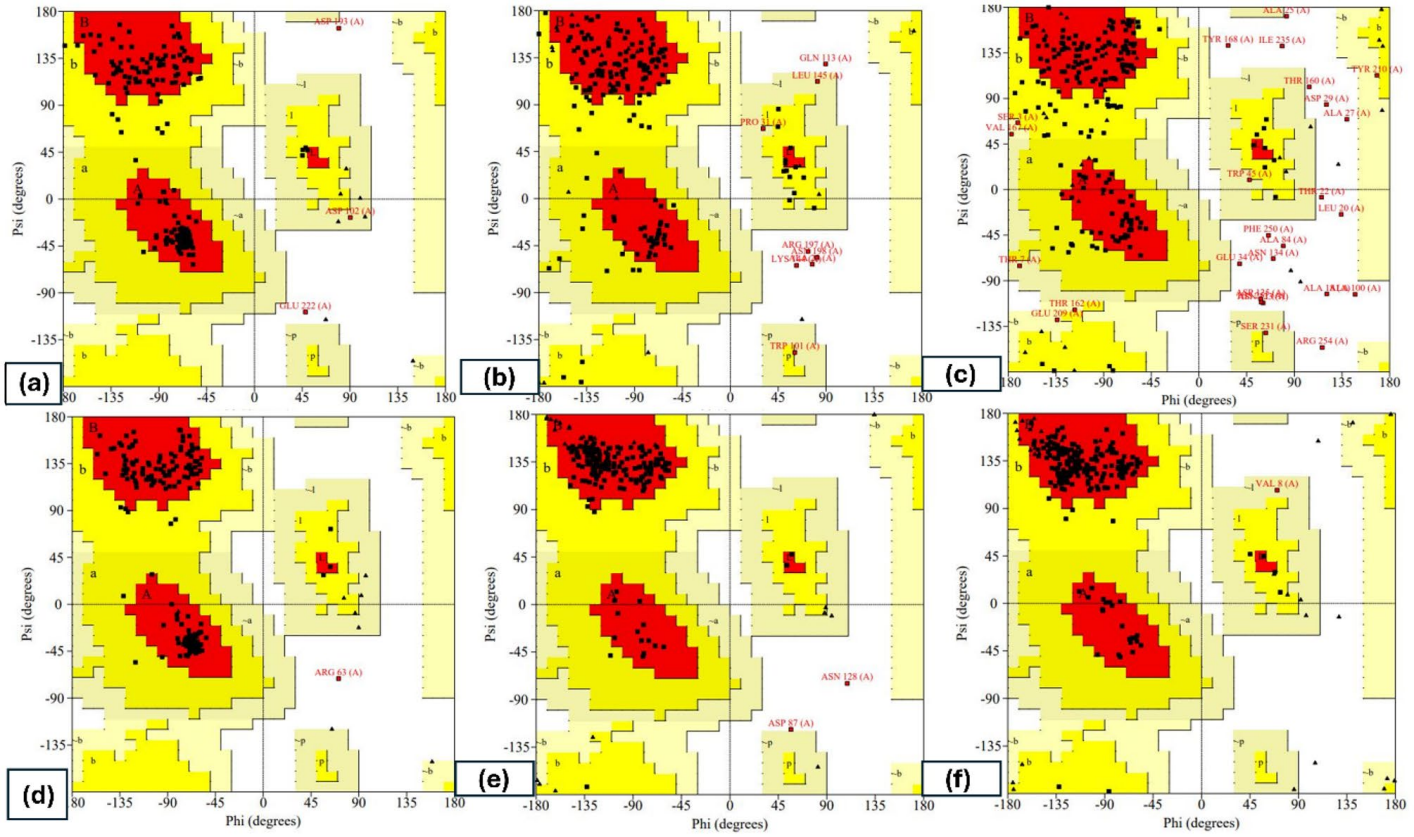
The Ramachandran plot of (**a**) BvrR protein of I-TASSER, (**b**) OMP25 protein of I-TASSER. (**c**) OMP31 of I-TASSER, (**d**) BvrR protein of AlphaFold, (**e**) OMP25 protein of AlphaFold, and (f) OMP31 protein of AlphaFold.

The best 3-D models of the correct fold were chosen through model evaluation out of all the feasible conformations that were the most similar to the original structure^18^. The results of the various evaluation servers' scores were summarized in (Tables 4, 5 and 6). The TM value of the selected modified model was higher than 0.5, suggesting high accuracy. If a value is between 0.5 and 0.17 or less than 0.17, this yields a model with a topology that is appropriately accurate whatever the size of the protein^14^. The QMEANDisCo score provides an estimate of the absolute quality of a model by relating it to reference structures solved by X-ray crystallography. It uses the single terms of QMEAN as a basis. All terms are combined using neural networks trained to predict per-residue lDDT scores in range [0, 1]^19^. According to the server section from CASP11 to CASP15 tests, I-TASSER and AlphaFold servers were positioned as the best approach. The I-TASSER server provides the best model according to RMSD (0.38, 0.75, and 0.66), TM-score (0.9957, 0.9892, and 0.9881), Z-score (17.139, 27.892, and 27.689), QMEAN (0.73, 0.48, and 0.62), ERRAT score (96.9298, 80.791, and 74.4589), and Ramachandran plot for the three target proteins, respectively (Fig. 1). The AlphaFold server provides the best model with RMSD (1.13, 1.94, and 2.42), TM-score (0.781, 0.880, and 0.906), Z-score (17.083, 27.892, and 27.700), QMEAN (0.75, 0.54 and 0.40), ERRAT score (95.9641, 86.5169 and 52.6316), and Ramachandran plot for the three target proteins, respectively (Fig. 1). These scores were chosen from the GalaxyWeb server as it has shown the best-refined results (Tables 4, 5 and 6).

### Motifs prediction

**Figure 2:**
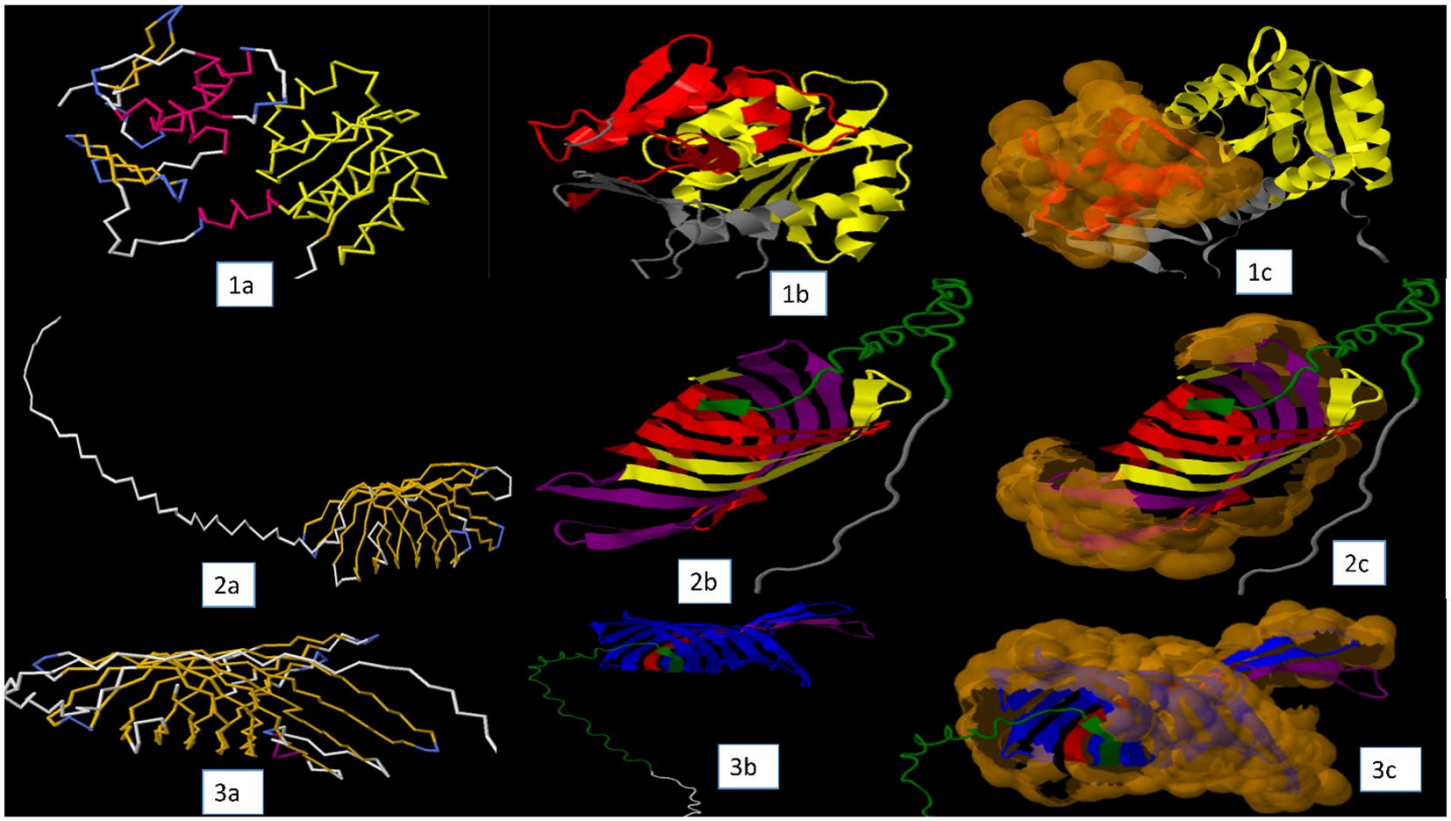
The best predicted three-dimensional structure of BvrR (1), OMP25 (2) and OMP31 (3) proteins. (**1a**) Backbone view of the best-predicted model. (**1b**) The cartoon view showed known and predicted motifs: The Response_reg (PF00072, 10..119) in yellow, Trans_reg_C (PF00486, 158..234) in red, and the rest of the predicted model in gray. (**1c**) Solvent-accessible surface view showed the exposed regions. (**2a**) Backbone view of best-predicted model. (**2b**) The cartoon view showed known and predicted motifs: OMP_b-brl (PF13505, 12..213) in green, OmpA_membrane (PF01389, 49..213) in red, Opacity (PF02462, 117..213) in yellow, Porin_2 (PF02530, 143..209) in purple, Surface_Ag_2 (PF01617, 128..190) in blue and the rest of predicted model in gray. (**2c**) Solvent-accessible surface view showed the exposed regions. (**3a**) Backbone view of best-predicted model. (**3b**) The cartoon view showed known and predicted motifs: OMP_b-brl (PF13505, 16..261) in green, OmpA_membrane (PF01389, 77..261) in red, Opacity (PF02462, 179..244) in yellow, Porin_2 (PF02530, 189..245) in purple, Autotransporter (PF03797, 52..222) in blue and the rest of predicted model in gray. (**3c**) Solvent-accessible surface view showed the exposed regions.

Using motifFinder, ScanProsite, and Motif Scan servers, the motifs in the BvrR, OMP25, and OMP31 proteins were analyzed and listed in (Tables 7 and 8). MotifFinder server showed that the BvrR protein has two motifs: Response_reg (PF00072, 10..119) and Trans_reg_C (PF00486, 158..234), OMP25 and OMP31 proteins have the same four motifs but in a different position: OMP_b-brl (PF13505, 12..213, 16..261), ompA_membrane (PF01389, 49..213, 77..261), opacity (PF02462, 117..213, 179..244), and Porin_2 (PF02530, 143..209, 189..245 ), respectively. Otherwise, there is one unique motif for each protein: surface_Ag_2 (PF01617, 128..190) for OMP25 protein and autotransporter (PF03797, 52..222) for OMP31 protein (Fig. 2). Furthermore, the ScanProsite server displayed two motifs for only BvrR protein: RESPONSE_REGULATORY (PS50110, 9..122) and OmpR/PhoB-type (PS51755, 137..236).

**Table 7:** Motif prediction of three target proteins using servers.

Category	Server	Protein	ID	Signature	Matching Position	E-value
DNA associated protein motif posttranslational modifications	MotifFinder	BvrR	Response_reg	PF00072, Response regulator receiver domain	10..119	1.2e–28
			Trans_reg_C	PF00486, Transcriptional regulatory protein, C terminal	158..234	3.7e–22
		OMP25	OMP_b-brl	PF13505, Outer membrane protein beta-barrel domain	12..213	7.3e–24
			OmpA_membrane	PF01389, OmpA-like transmembrane domain	49..213	3.4e–07
			Opacity	PF02462, Opacity family porin protein	117..213	0.00034
			Porin_2	PF02530, Porin subfamily	143..209	0.00072
			Surface_Ag_2	PF01617, Surface antigen	128..190	0.19
		OMP31	OMP_b-brl	PF13505, Outer membrane protein beta-barrel domain	16..261	2.8e–23
			OmpA_membrane	PF01389, OmpA-like transmembrane domain	77..261	1.8e–08
			Opacity	PF02462, Opacity family porin protein	179..244	1.3e–06
			Porin_2	PF02530, Porin subfamily	189..245	3.7e–05
			Autotransporter	PF03797, Autotransporter beta-domain	52..222	0.00021
	ScanProsite	BvrR	PS50110	RESPONSE_REGULATORY Response regulatory domain profile	9..122	–
			PS51755	OMPR_PHOB OmpR/PhoB-type DNA-binding domain profile	137..236	–

**Table 8:** Prediction of post-translation modification site of three target proteins using motif scan server.

Signature	Position of proteins		
	BvrR	OMP25	OMP31
CK2_PHOSPHO_SITE—Casein kinase II phosphorylation site	36–39	–	66–69
	42–45		136–139
	85–88		160–163
	164–167		183–186
MYRISTYL—N-myristoylation site	96–101	47–52	47–52
		77–82	89–94
	179–184	117–122	102–107
		137–142	130–135
			151–156
			172–177
			194–199
			207–212
PKC_PHOSPHO_SITE—Protein kinase C phosphorylation site	85–87	3–5	3–5
	110–112	68–70	115–117
	155–157	118–120	152–154
		195–197	162–164
			217–219
			252–254
BIG1—Big-1 (bacterial Ig-like domain 1) domain profile	1–13	–	–
RESPONSE_REGULATORY—Response regulatory domain profile	9–122	–	–
	10–119		
SRR—Seven residue repeat	212–225	–	–
Trans_reg_C—Transcriptional regulatory protein, C terminal	158–234	–	–
DUF1344—Protein of unknown function (DUF1344)	–	2–26	
OmpA_membrane—OmpA-like transmembrane domain	–	161–182	162–225
		8–213	26–261
Porin_2—Porin subfamily	–	158–209	8–51
		1–209	8–243
Opacity—Opacity family porin protein	–	97–213	93–261
			189–261
AMIDATION—Amidation site	–	–	227–230
ASN_GLYCOSYLATION—N-glycosylation site	–	–	134–137
			215–218
CBM_2—Cellulose binding domain	–	–	206–216

### Identification, annotation, and analysis of domain architectures

Using the superfamily server, the BvrR protein has two hits matched at 7..130 that are categorized in CheY-like superfamily and family. The other hit, located in 133..235, was classified as the C-terminal effector domain of the bipartite response regulators superfamily and PhoB-like family. On the other hand, OMP25 and OMP31 proteins have the same match in the OMPA-like superfamily and outer membrane protein family within different locations at 43..213 and 43..261, respectively (Table 9).

**Table 9:** Structural classification of three target proteins using superfamily server.

Protein	Domain	Region	Superfamily	Family	E-value
BvrR	1	7–130	CheY-like	CheY-related	0.00014
	2	133–235	The C-terminal effector domain of the bipartite response regulators	PhoB-like	0.0014
OMP25	1	43–213	OMPA-like	Outer membrane protein	0.0045
OMP31	1	43–261	OMPA-like	Outer membrane protein	0.005

Using CATH, the BvrR protein was classified as alpha–beta (3), 3-Layer (aba) Sandwich (3.40) architecture, Rossmann fold (3.40.50) topology, and response regulator (3.40.50.2300) homologous superfamily. On the other hand, there were no results matched for the OMP25 protein. The OMP31 protein was classified as Mainly Beta (2), Beta Barrel (2.40) architecture, Porin (2.40.160) topology, and (2.40.160.20) homologous superfamily (Table 10).

**Table 10:** Structural classification of three target proteins using the CATH Server.

Protein	Level	Description	Match	E-value
BvrR	Class	Alpha Beta (3)	Two-component system response regulator (3.40.50.2300/FF/328)	1.6e–23
	Architecture	3-Layer(aba) Sandwich (3.40)	Two-component system sensor histidine kinase/response regulator (3.40.50.2300/FF/138)	1.3e–25
	Topology	Rossmann fold (3.40.50)	DNA-binding transcriptional regulator NtrC (3.40.50.2300/FF/18)	6.2e–24
	Homologous superfamily	Response regulator (3.40.50.2300)	Response regulator GraR (3.40.50.2300/FF/232)	1.4e–21
			Sensory transduction histidine kinase (3.40.50.2300/FF/444)	3.7e–21
			Two-component system KDP operon response regulator KdpE (3.40.50.2300/FF/194)	6.7e–21
			Unplaced genomic scaffold supercont1.240, whole genome shotgun sequence (3.40.50.2300/FF/230)	1.0e–18
			Sporulation initiation phosphotransferase F (3.40.50.2300/FF/700)	3.9e–17
			Diguanylate cyclase response regulator (3.40.50.2300/FF/346)	6.3e–17
			Stress response regulator/HFS transcription factor (3.40.50.2300/FF/212)	9.4e–16
			Fis family transcriptional regulator (3.40.50.2300/FF/74)	1.2e–15
OMP25	–	–	–	–
OMP31	Class	Mainly Beta (2)	Putative outer membrane protein (2.40.160.20/FF/32)	5.3e–15
	Architecture	Beta Barrel (2.40)		
	Topology	Porin (2.40.160)		
	Homologous superfamily	2.40.160.20		

### Epitopes prediction

Linear B cell epitopes of the three target proteins of *Brucella* were done using SVMTriP, and Bepipred Linear Epitope Prediction 2.0 servers, while discontinuous B cell epitopes were identified using ElliPro (Tables 11, 12, 13 and 14). Additionally, Prediction of MHC-I or CD8 + T cell (HLA-A) and MHC-II or CD4 + T cell binding epitopes (HLA-DRB) for cytotoxic T lymphocyte (CTL) and T helper lymphocyte (THL), respectively, were performed using RANKPEP, SYFPEITHI and MHCII-NP servers (Tables 15 and 16). The antigenicity of epitopes was assayed using VaxiJen v2.0 and the allergenic and toxic probability of each epitope were evaluated using AllergenFP and ToxinPred servers. The epitopes with antigenicity higher than 0.4 and non-allergen were selected.

**Table 11:** B cell prediction of three target proteins using SVMTriP server.

Protein	Location	Epitope	Score	Antigenicity	Allergenicity	Toxicity
BvrR	44–63	LDGLMARPPNLAIFDIKMPR	0.430	1.2410	NON-ALLERGEN	Non-Toxin
OMP25	52–71	LYLGYGWNKAKTSTVGSIKP	1.000	0.6012	NON-ALLERGEN	Non-Toxin
	87–106	DQIVYGVEGDAGYSWAKKSK	0.947	0.6073	NON-ALLERGEN	Non-Toxin

**Table 12:** B cell prediction of three target proteins using bepipred linear epitope prediction 2.0.

Protein	Location	Peptide	Average	Minimum	Maximum	Antigenicity	Allergenicity	Toxicity
BvrR	125–139	VARRHAKPAGQQAKS	0.463	0.288	0.676	0.7755	NON-ALLERGEN	Non-Toxin
OMP25	26–46	AIQEQPPVPAPVEVAPQYSWA	0.510	0.243	0.656	0.7219	NON-ALLERGEN	Non-Toxin
	98–115	GYSWAKKSKDGLEVKQGF				0.9518	NON-ALLERGEN	Non-Toxin
	141–157	SQIKLNNGLDDESKFRV				0.8627	NON-ALLERGEN	Non-Toxin
	178–203	EYRYTQYGNKNYDLAGTTVRNKLDTQ				1.0258	NON-ALLERGEN	Non-Toxin
OMP31	29–45	DIIVAEPAPVAVDTFSW	0.530	0.252	0.725	0.9679	NON-ALLERGEN	Non-Toxin
	57–85	AGGKFKHPFSGIEQDGAQDFSGSLDVTAS				0.7610	NON-ALLERGEN	Non-Toxin
	111–144	FQGSTVKSKLVDNGDLSDIGVAGNLSGDESFVLE				0.6632	NON-ALLERGEN	Non-Toxin
	177–199	KVKTSLSAYDDGESFSAGNSKTK				0.9342	NON-ALLERGEN	Non-Toxin
	222–251	YLYTDLGKRSFNYIDEENVNINMENKVNFH				0.6510	NON-ALLERGEN	Non-Toxin

**Table 13:** ElliPro antibody epitope prediction of I-TASSER and AlphaFold models of target proteins.

Server	Protein	Location	Peptide	No. of residues	Score	Antigenicity	Allergenicity	Toxicity
I-TASSER	BvrR	85–94	TSKDDEIDEL	10	0.816	0.7267	NON-ALLERGEN	Non-Toxin
		208–237	KRLRKKFKAVDDSFEMIETLYGVGYRFREA	30	0.69	0.4274	NON-ALLERGEN	Non-Toxin
		193–205	DEQVYVDDRTIDS	13	0.674	0.8660	NON-ALLERGEN	Non-Toxin
		126–137	ARRHAKPAGQQA	12	0.652	0.6334	NON-ALLERGEN	Non-Toxin
	OMP25	102–114	AKKSKDGLEVKQG	13	0.788	1.8858	NON-ALLERGEN	Non-Toxin
		142–155	QIKLNNGLDDESKF	14	0.785	0.7871	NON-ALLERGEN	Non-Toxin
		59–73	NKAKTSTVGSIKPDD	15	0.748	0.7941	NON-ALLERGEN	Non-Toxin
		23–47	AADAIQEQPPVPAPVEVAPQYSWAG	25	0.746	0.5963	NON-ALLERGEN	Non-Toxin
		182–200	TQYGNKNYDLAGTTVRNKL	19	0.669	0.8759	NON-ALLERGEN	Non-Toxin
	OMP31	177–198	KVKTSLSAYDDGESFSAGNSKT	22	0.823	0.8254	NON-ALLERGEN	Non-Toxin
		58–82	GGKFKHPFSGIEQDGAQDFSGSLDV	25	0.79	0.6199	NON-ALLERGEN	Non-Toxin
		116–143	VKSKLVDNGDLSDIGVAGNLSGDESFVL	28	0.786	0.5532	NON-ALLERGEN	Non-Toxin
		28–46	ADIIVAEPAPVAVDTFSWT	19	0.715	0.9214	NON-ALLERGEN	Non-Toxin
		209–216	EYAVTNNW	8	0.585	0.9221	NON-ALLERGEN	Non-Toxin
AlphaFold	OMP25	183–202	QYGNKNYDLAGTTVRNKLDT	20	0.671	0.9411	NON-ALLERGEN	Non-Toxin
		147–159	NGLDDESKFRVGW	13	0.659	0.9067	NON-ALLERGEN	Non-Toxin
	OMP31	1–39	MFSLKGTVMKTALLASVAMLFTSSAMAADIIVAEPAPVA	39	0.823	0.4288	NON-ALLERGEN	Non-Toxin
		119–142	KLVDNGDLSDIGVAGNLSGDESFV	24	0.642	0.5128	NON-ALLERGEN	Non-Toxin

**Table 14:** Predicted discontinuous epitope using ElliPro server.

Server	Protein	Residues	No. of residues	Score
I-TASSER	BvrR	A:M1, A:K2, A:E3, A:A4, A:S5, A:A6, A:T7, A:Q8, A:D14, A:D15, A:D16, A:R17, A:N18, A:I19, A:L20, A:T21, A:S22, A:S24, A:I25, A:A26, A:E28, A:S29, A:E30, A:G31, A:Y32, A:T38, A:D39, A:I59, A:R63, A:M64, A:T85, A:S86, A:K87, A:D88, A:D89, A:E90, A:I91, A:D92, A:E93, A:L94, A:T106, A:K107, A:P108, A:F109, A:S110, A:Q111, A:R112, A:L113, A:V115, A:E116, A:K119, A:A120, A:R123, A:A126, A:R127, A:R128, A:H129, A:A130, A:K131, A:P132, A:A133, A:G134, A:Q135, A:Q136, A:A137A:M1, A:K2, A:E3, A:A4, A:S5, A:A6, A:T7, A:Q8, A:D14, A:D15, A:D16, A:R17, A:N18, A:I19, A:L20, A:T21, A:S22, A:S24, A:I25, A:A26, A:E28, A:S29, A:E30, A:G31, A:Y32, A:T38, A:D39, A:I59, A:R63, A:M64, A:T85, A:S86, A:K87, A:D88, A:D89, A:E90, A:I91, A:D92, A:E93, A:L94, A:T106, A:K107, A:P108, A:F109, A:S110, A:Q111, A:R112, A:L113, A:V115, A:E116, A:K119, A:A120, A:R123, A:A126, A:R127, A:R128, A:H129, A:A130, A:K131, A:P132, A:A133, A:G134, A:Q135, A:Q136, A:A137	65	0.699
		A:E141, A:G143, A:Q144, A:L145, A:V146, A:T155, A:W156, A:K157, A:G158, A:E159, A:P160, A:V161, A:T162, A:L163, A:K213, A:F214	16	0.688
		A:R177, A:P178, A:G179, A:V180, A:V181, A:K182, A:S183, A:R184, A:D185, A:D193, A:E194, A:Q195, A:V196, A:Y197, A:V198, A:D199, A:D200, A:R201, A:T202, A:D204, A:S205, A:K208, A:R209, A:R211, A:K212, A:K215, A:A216, A:V217, A:D218, A:D219, A:S220, A:F221, A:E222, A:M223, A:I224, A:E225, A:T226, A:L227, A:Y228, A:G229, A:V230, A:G231, A:Y232, A:R233, A:F234, A:R235, A:E236, A:A237	48	0.677
	OMP25	A:T63, A:S64, A:T65, A:V66, A:G67, A:S68, A:I69, A:K70, A:P71, A:D72, A:D73, A:W74, A:A102, A:K103, A:K104, A:S105, A:K106, A:D107, A:G108, A:L109	20	0.786
		A:E110, A:V111, A:K112, A:Q113, A:G114, A:Q142, A:I143, A:K144, A:L145, A:N146, A:N147, A:G148, A:L149, A:D150, A:D151, A:E152, A:S153, A:K154	18	0.783
		A:I27, A:Q28, A:E29, A:Q30, A:P31, A:P32, A:V33, A:P34, A:A35, A:P36, A:V37, A:E38, A:V39, A:A40, A:P41, A:Q42, A:S44, A:W45, A:A46, A:G47, A:N83, A:F84, A:Q85, A:Q86, A:D87, A:Q88, A:I89, A:V90	28	0.716
		A:T182, A:Q183, A:Y184, A:G185, A:N186, A:K187, A:N188, A:D190, A:L191, A:A192, A:G193, A:T194, A:T195, A:V196, A:R197, A:N198, A:K199, A:L200, A:D201	19	0.657
		A:W58, A:N59, A:K60, A:A61, A:K62	5	0.577
		A:I9, A:V10, A:S11, A:A12, A:A13, A:L14, A:L15, A:P16, A:F17, A:S18, A:A19, A:T20, A:A21, A:F22, A:A23, A:A24, A:D25, A:A26, A:D126, A:L127, A:N128, A:P129, A:K168, A:L169, A:T170, A:D171, A:N172, A:I173, A:Y211, A:K212	30	0.521
	OMP31	A:F65, A:S66, A:G67, A:I68, A:E69	5	0.969
		A:Q70, A:D71, A:G72, A:A73, A:Q74, A:D75	6	0.898
		A:G176, A:V178, A:K179, A:T180, A:S181, A:L182, A:S183, A:A184, A:Y185, A:D187, A:G188, A:E189, A:S190, A:F191, A:S192, A:A193, A:G194, A:N195, A:S196, A:K197, A:T198	21	0.812
		A:K62, A:H63, A:P64	3	0.806
		A:T115, A:V116, A:K117, A:S118, A:K119, A:L120, A:V121, A:D122, A:N123, A:G124, A:D125, A:L126, A:S127, A:D128, A:I129, A:G130, A:V131, A:A132, A:G133, A:N134, A:L135, A:S136, A:G137, A:D138, A:E139, A:S140, A:F141, A:V142, A:L143	29	0.77
		A:F76, A:S77, A:G78, A:S79, A:L80, A:D81, A:V82	7	0.666
		A:G58, A:G59, A:K60, A:F61	4	0.613
		A:L227, A:G228, A:K229, A:R230, A:S231, A:F232, A:N233, A:Y234, A:I235, A:E237, A:E238, A:N239, A:V240, A:N241, A:I242, A:N243, A:M244, A:E245	18	0.598
		A:M1, A:F2, A:S3, A:L4, A:G6, A:T7, A:V8, A:T11, A:A12, A:L13, A:L14, A:M19, A:L20, A:F21, A:A27, A:A28, A:D29, A:I30, A:I31, A:V32, A:A33, A:E34, A:P35, A:A36, A:P37, A:V38, A:A39, A:V40, A:D41, A:T42, A:F43, A:S44, A:T46, A:W97, A:Q98, A:L99, A:A100, A:N101, A:G102, A:L103, A:V104, A:L105, A:G158, A:F159, A:T160, A:P161, A:T162, A:E163, A:R164, A:L165, A:M166, A:E209, A:Y210, A:A211, A:V212, A:T213, A:N214, A:N215, A:Y259, A:K260	60	0.575
AlphaFold	BvrR	A:G143, A:Q144, A:W156, A:K157	4	0.771
		A:A130, A:K131, A:P132, A:A133, A:G134, A:Q135, A:Q136, A:A137, A:K138, A:S139, A:E141, A:R142, A:V146, A:Q149, A:E150, A:R151	16	0.742
		A:G158, A:E159, A:P160, A:Q176, A:R177, A:P178, A:G179, A:V180, A:V181, A:K182, A:S183, A:R184, A:D185, A:V198, A:D199, A:D200, A:R201, A:K215, A:A216, A:V217, A:D218, A:D219, A:S220, A:F221, A:E222, A:M223, A:E225, A:T226, A:L227, A:Y228, A:G229, A:V230, A:G231, A:R233, A:R235, A:E236, A:A237	37	0.731
		A:E3, A:A4, A:S5, A:A6, A:T7, A:Q8, A:D14, A:D15, A:D16, A:N18, A:I19, A:L20, A:T21, A:S22, A:S24, A:I25, A:A26, A:E28, A:S29, A:E30, A:G31, A:Y32, A:R33, A:E35, A:T36, A:T38, A:D39, A:G40, A:A41, A:S42, A:L44, A:D45, A:G46, A:M48, A:A49, A:R50, A:M61, A:P62, A:R63, A:M64, A:D65, A:E68, A:R72, A:Q75, A:K76, A:S86, A:K87, A:D88, A:D89, A:I91, A:K107, A:P108, A:F109, A:S110, A:Q111	55	0.69
	OMP25	A:M1, A:R2, A:T3, A:L4, A:K5, A:S6, A:L7, A:V8, A:I9, A:V10, A:S11, A:A12, A:A13, A:L14, A:L15, A:P16, A:F17, A:S18, A:A19, A:T20	20	0.947
		A:A21, A:F22, A:A23, A:A24, A:D25, A:A26, A:I27, A:Q28	8	0.872
		A:K60, A:K62, A:T63, A:S64, A:T65, A:V66, A:G67, A:S68, A:K106, A:D107, A:G108, A:N147, A:G148, A:L149, A:D150, A:D151, A:E152, A:S153, A:K154, A:W159, A:Q183, A:G185, A:N186, A:K187, A:N188, A:Y189, A:D190, A:L191, A:A192, A:G193, A:T194, A:T195, A:V196, A:R197, A:N198, A:K199, A:L200, A:D201	38	0.706
		A:Q30, A:P31, A:P32	3	0.613
		A:Y43, A:T50, A:L52, A:N128, A:P129, A:V130, A:A167, A:L169, A:T170, A:D171, A:N172, A:I173, A:V177, A:V207, A:G208, A:I209, A:G210, A:Y211, A:K212	19	0.613
		A:Y56, A:W58, A:W74, A:Q203	4	0.587
	OMP31	A:M1, A:F2, A:S3, A:L4, A:K5, A:G6, A:T7, A:V8, A:M9, A:K10, A:T11	11	0.972
		A:A12, A:L13, A:L14, A:A15, A:S16, A:V17, A:A18, A:M19, A:L20, A:F21	10	0.938
		A:F65, A:S66, A:G67, A:I68, A:E69, A:Q70, A:D71, A:G72, A:A73, A:Q74, A:D75, A:F76, A:S77, A:G78, A:G228, A:K229, A:R230, A:S231, A:F232, A:N233, A:Y234, A:I235, A:D236, A:E237, A:E238, A:N239, A:V240, A:N241, A:I242, A:N243, A:E245, A:K247	32	0.753
		A:S23, A:S24, A:A25, A:M26, A:A27, A:A28, A:D29, A:I30, A:V32, A:A33, A:E34, A:P35, A:A36	13	0.715
		A:K119, A:V121, A:D122, A:N123, A:G124, A:D125, A:L126, A:S127, A:D128, A:V131, A:A132, A:G133, A:N134, A:L135, A:S136, A:G137, A:D138, A:E139, A:S140, A:V142, A:Q148, A:K177, A:S183, A:A184, A:Y185, A:D186, A:D187, A:G188, A:E189, A:S190, A:F191, A:S192, A:A193, A:G194, A:N195, A:S196, A:K197	37	0.687
		A:Y56, A:F87, A:T115	3	0.609
		A:I52, A:R164, A:Y210, A:V212, A:T213, A:N215, A:W216, A:G256, A:L257, A:N258, A:Y259	11	0.564
		A:L99, A:A100, A:N101, A:G102, A:F159, A:P161, A:T162	7	0.531

**Table 15:** The CD8 + T cell epitopes of the three target proteins using different servers.

Servers	Protein	Allele	RANK	POS	SEQUENCE	SCORE	Antigenicity	Allergenicity	Toxicity
RANKPEP	OMP25	HLA_A01	1	181	YTQYGNKNY	16.517	0.5172	NON-ALLERGEN	Non-Toxin
			2	91	YGVEGDAGY	12.453	1.7710	NON-ALLERGEN	Non-Toxin
			3	176	RVEYRYTQY	5.577	1.3330	NON-ALLERGEN	Non-Toxin
			4	159	WTAGAGLEA	5.331	1.4452	NON-ALLERGEN	Non-Toxin
		HLA_A0201	1	137	GIAGSQIKL	70	1.8042	NON-ALLERGEN	Non-Toxin
			2	61	AKTSTVGSI	56	0.6583	NON-ALLERGEN	Non-Toxin
	OMP31	HLA_A01	1	167	VYGTGGLAY	10.33	0.4684	NON-ALLERGEN	Non-Toxin
			2	108	EADFQGSTV	7.767	1.1171	NON-ALLERGEN	Non-Toxin
		HLA_A0201	1	3	SLKGTVMKT	74	1.0532	NON-ALLERGEN	Non-Toxin
Syfpeithi	BvrR	HLA-A*01	1	189	DAAYDEQVY	16	0.6631	NON-ALLERGEN	Non-Toxin
		HLA-A*02:01	1	173	SLAQRPGVV	24	0.4381	NON-ALLERGEN	Non-Toxin
			2	202	TIDSHIKRL	21	1.0243	NON-ALLERGEN	Non-Toxin
	OMP25	HLA_A01	1	176	RVEYRYTQY	23	1.3330	NON-ALLERGEN	Non-Toxin
			2	181	YTQYGNKNY	23	0.5172	NON-ALLERGEN	Non-Toxin
			3	91	YGVEGDAGY	17	1.7710	NON-ALLERGEN	Non-Toxin
		HLA_A0201	1	137	GIAGSQIKL	25	1.8042	NON-ALLERGEN	Non-Toxin
			2	130	VMPYLTAGI	22	0.4643	NON-ALLERGEN	Non-Toxin
			3	161	AGAGLEAKL	21	2.4411	NON-ALLERGEN	Non-Toxin
	OMP31	HLA_A01	1	216	WTLKSEYLY	22	1.1851	NON-ALLERGEN	Non-Toxin
			2	167	VYGTGGLAY	20	0.4684	NON-ALLERGEN	Non-Toxin
			3	214	NNWTLKSEY	17	1.4935	NON-ALLERGEN	Non-Toxin
		HLA_A0201	1	3	SLKGTVMKT	23	1.0532	NON-ALLERGEN	Non-Toxin

**Table 16:** The CD4 + T cell epitopes of the three target proteins using the RANKPEP server.

Protein	Allele	RANK	POS	SEQUENCE	SCORE	Antigenicity	Allergenicity	Toxicity
BvrR	HLA-DRB * 0701	1	150	ERHTCTWKG	10.821	0.5180	NON-ALLERGEN	–
		2	163	LTVTEFLIL	6.347	1.0405	NON-ALLERGEN	–
	HLA-DRB * 0901	1	202	TIDSHIKRL	7.329	1.0243	NON-ALLERGEN	–
		2	57	FDIKMPRMD	7.312	2.2813	NON-ALLERGEN	–
		3	189	DAAYDEQVY	5.269	0.6631	NON-ALLERGEN	–
OMP25	HLA-DRB * 0701	1	79	FAGWNFQQD	18.575	1.0670	NON-ALLERGEN	–
		2	189	YDLAGTTVR	5.395	1.5360	NON-ALLERGEN	–
	HLA-DRB * 0901	1	115	FEGSLRARV	16.405	0.8448	NON-ALLERGEN	–
		2	189	YDLAGTTVR	10.014	1.5360	NON-ALLERGEN	–
		3	181	YTQYGNKNY	9.05	0.5172	NON-ALLERGEN	–
OMP31	HLA-DRB * 0701	1	39	AVDTFSWTG	13.766	0.5343	NON-ALLERGEN	–
		2	245	ENKVNFHTV	9.507	0.8534	NON-ALLERGEN	–
	HLA-DRB * 0901	1	216	WTLKSEYLY	6.98	1.1851	Non-ALLERGEN	–

SVMTriP and Bepipred 2.0 servers clarified that (1, 2, and 0) and (1, 4, and 5) dominant B cell epitopes for the BvrR, OMP25, and OMP31 proteins, respectively (Tables 11 and 12). On the other hand, the Ellipro server detected (4, 5, and 5) and (0, 2, and 2) dominant linear B cell epitopes for the BvrR, OMP25, and OMP31 proteins produced from I-TASSER and AlphaFold servers, respectively (Table 13). The prediction of discontinuous B cell epitopes using the Ellipro server revealed the presence of 129, 120, and 163 total residues for BvrR, OMP25, and OMP31 proteins of the I-TASSER model, and their average scores were 0.688, 0.673, and 0.745, respectively. The AlphaFold model had 112, 92, and 124 total residues, and their average scores were 0.733, 0.723, and 0.599 for the BvrR, OMP25, and OMP31 proteins, respectively (Table 14).

For CD8 + T cell epitope prediction, 9-mer alleles (HLA_A01 and HLA_A0201) were used. Through the RankPep server, the BvrR protein was found to have one CD8 + T cell epitope at position 173..181 (SLAQRPGVV) only in the case of the HLA_A0201 allele. OMP25 protein showed four and two CD8 + T cell epitopes, and OMP31 protein displayed two CD8 + T cell epitopes for each allele, respectively. Furthermore, using the Syfpeithi server clarified one and two CD8 + T cell epitopes of BvrR protein, three CD8 + T cell epitopes of OMP25 protein, and three and one CD8 + T cell epitopes of OMP31 protein for each allele, respectively **(**Table 15).

For CD4 + T cell epitope prediction, the RankPep server used two 9-mer alleles (HLA-DRB * 0701 and HLA-DRB * 0901). BvrR protein detected two and three CD4 + T cell epitopes, OMP25 protein showed two and three CD4 + T cell epitopes, and OMP31 protein displayed two and one CD4 + T cell epitopes for each allele, respectively (Table 16). Moreover, the MHCII-NP server predicted three, four, and three CD4 + T cell epitopes for the three target proteins, respectively (Table 17).

**Table 17:** MHCII-NP prediction for the three target proteins.

Protein	Location	Peptide	N motif	C motif	Cleavage probability score	Cleavage probability percentile rank	Antigenicity	Allergenicity	Toxicity
BvrR	50–63	RPPNLAIFDIKMPR	ARP	PRM	1.23746	0.00	1.4171	NON-ALLERGEN	Non-Toxin
	39–53	DGASALDGLMARPPN	TDG	PNL	0.81440	0.13	0.6988	NON-ALLERGEN	Non-Toxin
	193–208	DEQVYVDDRTIDSHIK	YDE	IKR	0.79870	0.17	1.0873	NON-ALLERGEN	Non-Toxin
OMP25	95–108	GDAGYSWAKKSKDG	EGD	DGL	0.87185	0.00	0.7750	NON-ALLERGEN	Non-Toxin
	128–142	NPVMPYLTAGIAGSQ	LNP	SQI	0.74398	0.09	0.6498	NON-ALLERGEN	Non-Toxin
	70–85	KPDDWKAGAFAGWNFQ	IKP	FQQ	0.74048	0.14	0.7280	NON-ALLERGEN	Non-Toxin
	107–122	DGLEVKQGFEGSLRAR	KDG	ARV	0.72066	0.19	1.4627	NON-ALLERGEN	Non-Toxin
OMP31	242–256	INMENKVNFHTVRLG	NIN	LGL	0.75919	0.04	0.7496	NON-ALLERGEN	Non-Toxin
	240–254	VNINMENKVNFHTVR	NVN	VRL	0.61905	0.08	1.0609	NON-ALLERGEN	Non-Toxin
	109–123	ADFQGSTVKSKLVDN	EAD	DNG	0.60720	0.11	1.0226	NON-ALLERGEN	Non-Toxin

The final result from all previous servers for B and T cell epitopes prediction overlapped to conclude that there were (5, 6, and 5) dominant B-cell epitopes, (3, 4, and 4) dominant CTL epitopes, and (4, 5 and 4) dominant THL epitopes for the three target proteins (BvrR, OMP25, and OMP31), respectively (Table 18).

**Table 18:** The final overlapped B and T cells epitopes from the different servers used.

Epitopes	BvrR		OMP25		OMP31	
	Position	Sequence	Position	Sequence	Position	Sequence
B-cell	44–63	LDGLMARPPNLAIFDIKMPR	26–46	AIQEQPPVPAPVEVAPQYSWA	29–45	DIIVAEPAPVAVDTFSW
	126–137	ARRHAKPAGQQA	52—71	LYLGYGWNKAKTSTVGSIKP	58–82	GGKFKHPFSGIEQDGAQDFSGSLDV
	85–93	TSKDDEIDEL	98—106	GYSWAKKSK	119–142	KLVDNGDLSDIGVAGNLSGDESFV
	193–205	DEQVYVDDRTIDS	102–114	AKKSKDGLEVKQG	177–198	KVKTSLSAYDDGESFSAGNSKT
	208–237	KRLRKKFKAVDDSFEMIETLYGVGYRFREA	142–155	QIKLNNGLDDESKF	222–251	YLYTDLGKRSFNYIDEENVNINMENKVNFH
			183–200	QYGNKNYDLAGTTVRNKL		
CTL	173–181	SLAQRPGVV	61–69	AKTSTVGSI	3–11	SLKGTVMKT
	189–197	DAAYDEQVY	91–99	YGVEGDAGY	24–32	SAMAADIIV
	202–210	TIDSHIKRL	159–167	WTAGAGLEA	167–175	VYGTGGLAY
			181–189	YTQYGNKNY	216–224	WTLKSEYLY
THL	39–53	DGASALDGLMARPPN	79–87	FAGWNFQQD	39–47	AVDTFSWTG
	57–65	FDIKMPRMD	95–108	GDAGYSWAKKSKDG	109–123	ADFQGSTVKSKLVDN
	150–158	ERHTCTWKG	115–123	FEGSLRARV	216–224	WTLKSEYLY
	163–171	LTVTEFLIL	128–142	NPVMPYLTAGIAGSQIKL	245–253	ENKVNFHTV
			189–197	YDLAGTTVR		

### Vaccine construction and validation

**Figure 3:**
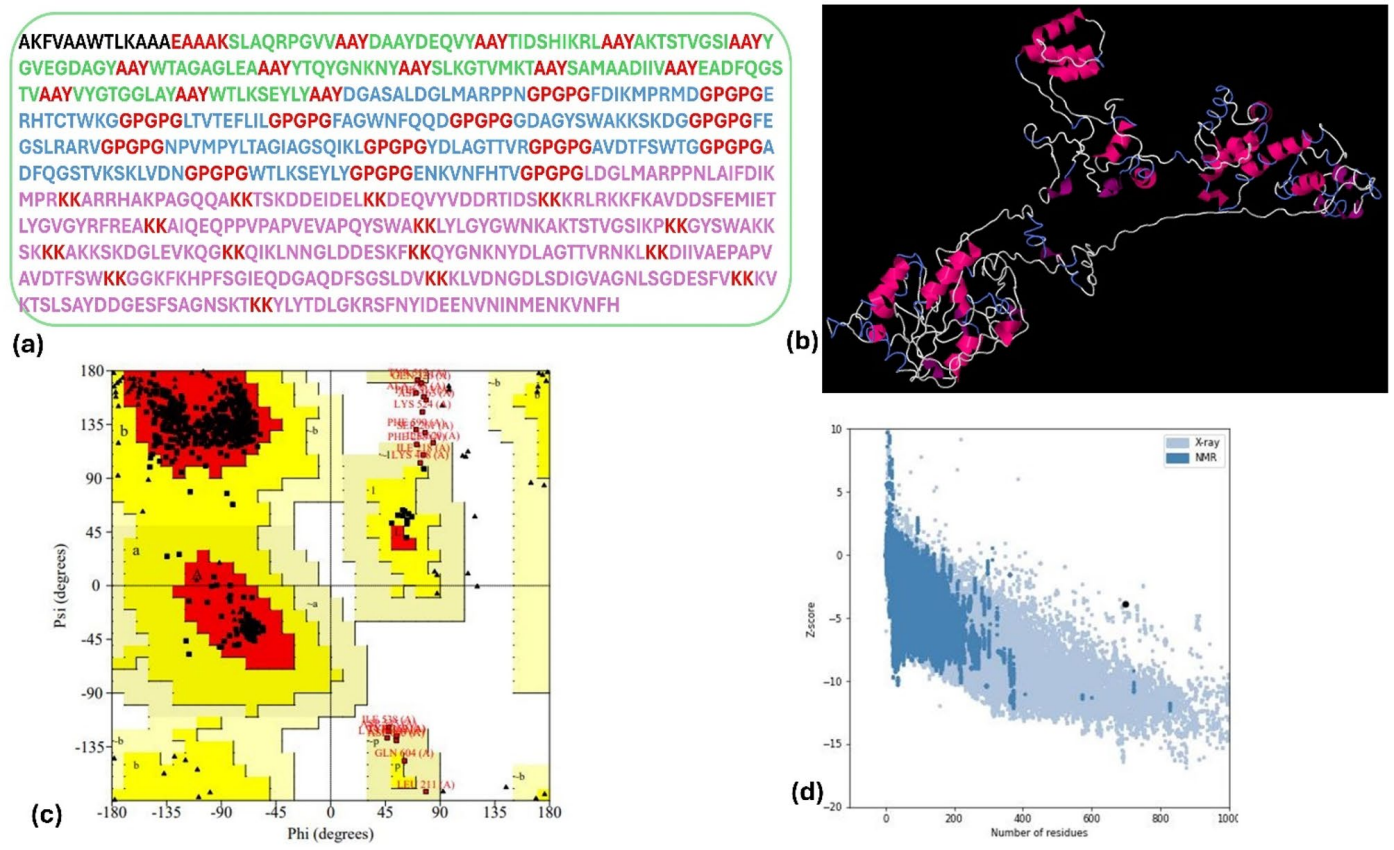
Constructed vaccine candidate against BvrR, OMP25, and OMP3 receptor *brucella*. (**a**) The designed vaccine candidate is sequentially like the PADRE sequence (Black color), followed by the next 5 amino acids of EAAAK linker (Red color), then CTL epitopes (Green color) from 19 to 159, then HTL epitopes (Blue color) from 163 to 365, then B-cell epitopes (Purple color) from 371 to 698. All the linkers have been represented in red color. (**b**) The 3D cartoon view of the designed vaccine. (**c**) The Ramachandran plot of the designed vaccine. (**d**) Z score of the designed vaccine.

The chimeric vaccine for the three target proteins was constructed using the final overlapped epitopes from the different servers and linkers. It consists of (3, 4, and 4) CTL epitopes, connected by AAY linkers, (4, 5, and 4) THL epitopes, connected by GPGPG linkers, and (5, 6, and 5) B-cell epitopes connected by KK linkers (Fig. 3a). The final constructed vaccine length was 698 amino acids. The antigenicity of this designed vaccine was 0.8942 and it is non-allergen and nontoxic.

The physiochemical properties of the constructed vaccine using ProtParam determined that it contains 698 amino acids and the molecular weight was found to be 74,676.32 Da. The theoretical isoelectric point (PI) was obtained as 9.36 specifies it as positive as an isoelectric point above 7.0 shows positively charged proteins. Instability-Index (II) was 24.79; which categorized our vaccine as a stable one. The aliphatic index was 63.01 indicating that the vaccine is more thermostable and can withstand high temperatures. A total number of carbon (C), hydrogen (H), nitrogen (N), oxygen (O), and sulfur (S) were entitled by formula C_3367_H_5188_N_900_O_1003_S_11_. The Grand average of hydropathicity (GRAVY) was -0.561 which indicates this vaccine is more hydrophilic.

### Secondary structure prediction

The SOPMA and PredictProtein servers predicted the designed vaccine consisting of 29.37% alpha helix, 21.63% beta-sheet, 39.40% random coil, 47.28% exposed residues, and 52.71% buried amino acid.

### Tertiary structure prediction, refinement and validation of the constructed vaccine

I-TASSER, AlphaFold colab2, and Swiss model were utilized to predict the 3-D structure of the chimeric vaccine (Fig. 3b). The best model was chosen to refine via the GalaxyRefine tool which provided five models. The best-refined model of I-TASSER had TM-score (0.89), RMSD (0.62), GDT-HA (0.8764), MolProbity (2.89), clash score (13.91), Ramachandran Favored (81%), QMEAN (0.32), ERRAT score (75.8), Ramachandran plot (Fig. 3c), and Z score (Fig. 3d).

### Molecular docking

#### Virtual screening and molecular docking analysis

The MOE docking protocol was used, and parameters were set for docking initial hits into the binding pocket of *Brucella’s* three proteins (BvrR, OMP25, and OMP31). Based on docking score, binding energy, and binding affinity calculation, which indicated the effectiveness of these compounds, the top-ranked 20 compounds from lead-like ZINC 20 database and DrugBank database were selected as promising hits for evaluation and revealing significant interactions with most of the important binding pocket residues. For the BvrR protein of the best I-TASSER model, the top-ranked compounds interacted with the following residues: MET1, LYS2, GLU3, ALA4, THR7, GLN8, THR9, LEU47, MET48, PRO51, PRO52, ASN53, ARG74, LYS76, SER77, ASP78, LEU79, PRO80, ASP102, VAL121, ARG124, VAL125, ALA126, ARG127, ARG128, HIS129, ALA130, LYS131, PRO132, ALA133, GLY134, GLN135, GLN136, ALA137, LYS138, GLU150, and ARG151. For OMP25 protein of the best I-TASSER model, the top-ranked compounds interacted with the following residues: GLY57, TRP58, ASN59, LYS60, ASP73, LYS75, ALA76, ALA97, GLY98, TYR99, SER100, TRP101, ALA102, LYS104, SER105, ASP107, LEU109, GLU110, VAL111, GLN113, GLY114, PHE115, GLU116, ALA139, SER141, GLN142, ILE143, LYS144, LEU149, ASP150, ARG156, VAL157, GLY158, TRP159, THR160, ARG180, THR182, TYR184, LEU200, THR202, GLN203, ASP204, ILE205, and ARG206. For the OMP31 protein of the best I-TASSER model, the top-ranked compounds interacted with the following residues: GLY55, TYR56, ALA57, LYS60, VAL82, THR83, ALA84, SER85, GLY86, PHE87, VAL88, GLY89, GLY90, VAL91, PHE111, GLN112, GLY113, SER114, THR115, LEU143, GLU144, THR145, LYS146, VAL147, GLN148, TRP149, PHE150, THR152, ARG154, ALA174, LYS177, LYS197, THR198, LYS199, GLY201, TRP202, THR203, LEU223, THR225, ASP226, ASN246, LYS247, VAL248, ASN249, PHE250, HIS251, THR252, VAL253, and ARG254.

#### Binding energy and binding affinity calculations

**Figure 4:**
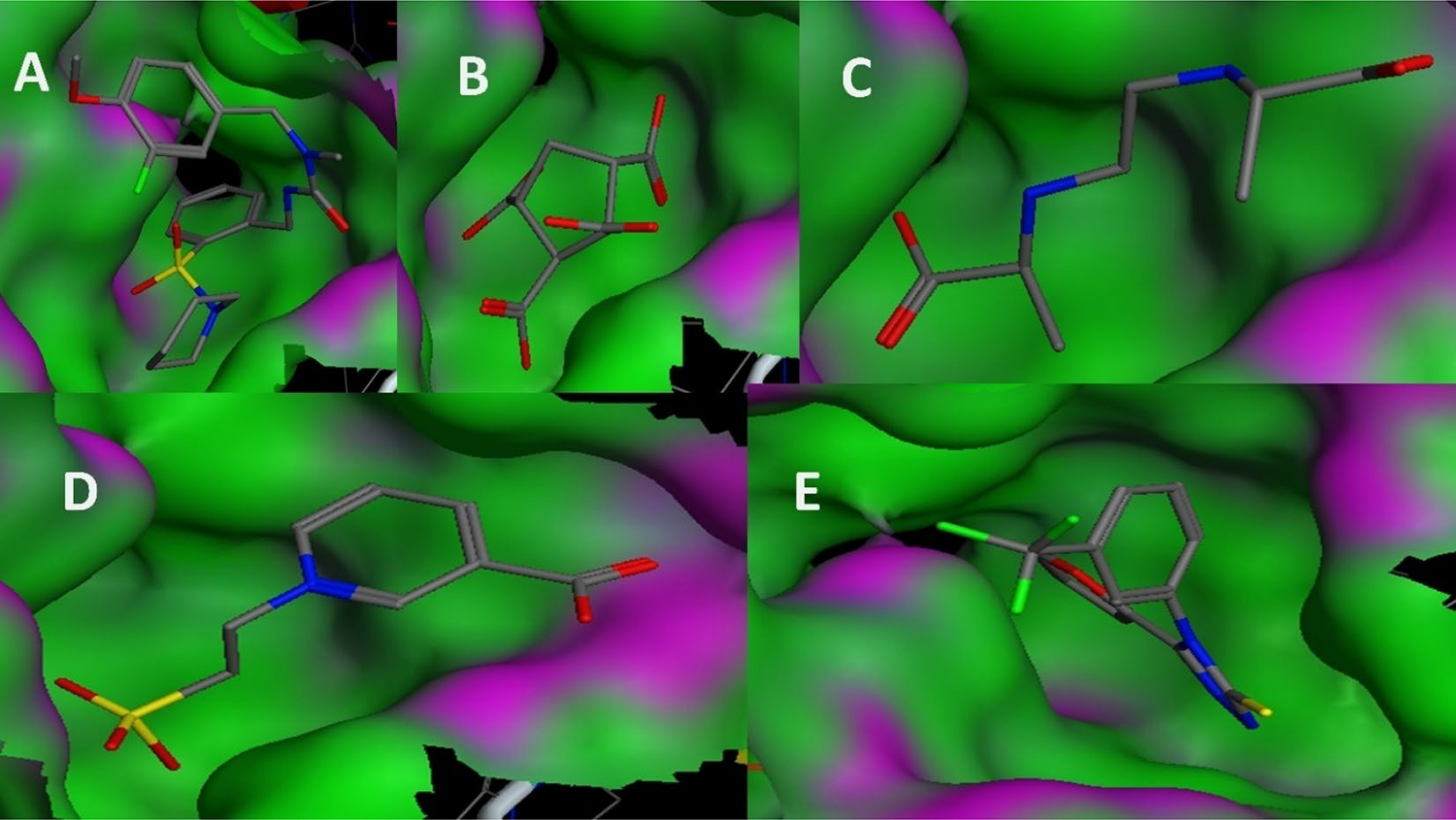
The docking binding mode of BvrR protein and ZINC database. (**A**) ZID 022035210, (**B**) ZID 00395425, (**C**) ZID 019366239, (**D**) ZID 01695129, and (**E**) ZID 00168942.

**Figure 5:**
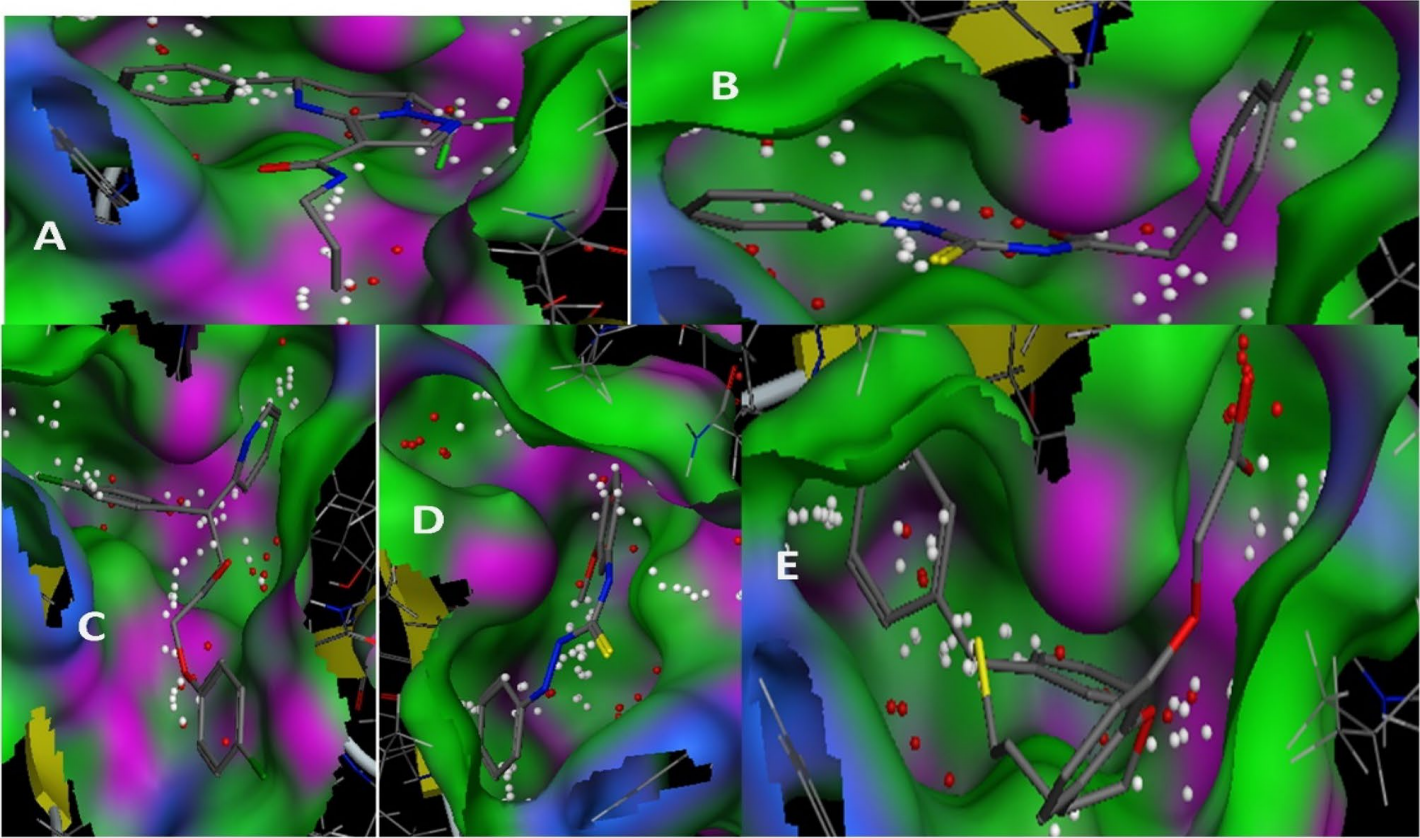
The docking binding mode of OMP25 protein and ZINC database. (**A**) ZID 02756403, (**B**) ZID 0486383, (**C**) ZID 09172386, (**D**) ZID 00172013, and (**E**) ZID 00602468.

**Figure 6:**
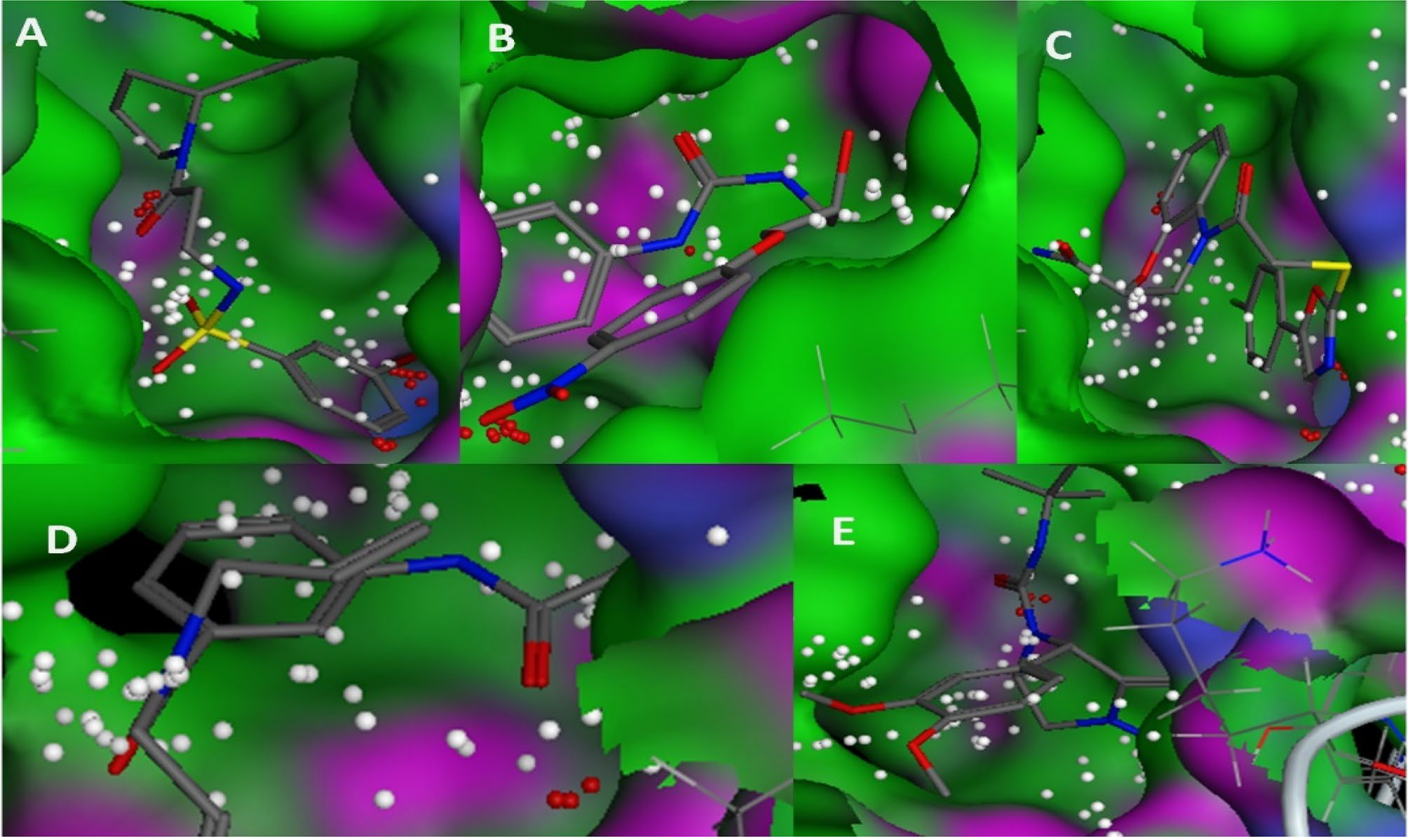
The docking binding mode of OMP31 protein and ZINC database (**A**) ZID 22551309, (**B**) ZID 00154214, (**C**) ZID 10847947, (**D**) ZID 00463723, and (**E**) ZID 04854502.

It is clear from (Tables 19, 20 and 21) that the best-predicted lead compound is ZINC ID (022035210), which possessed the docking score (− 16.8744), the binding energy (− 13.1624), and the binding affinity (− 58.3099) for BvrR protein. ZINC ID (02756403) had the docking score, (− 16.0424), the binding energy (− 14.1846), and the binding affinity (− 61.8023) for OMP25, and ZINC ID (22551309) had the docking score (− 14.7566), the binding energy (− 11.3077), and the binding affinity (− 57.0108) for OMP31 protein. Otherwise, (Supplementary Tables 1–3) showed that DrugBank ID (DB01901) possessed the docking score (− 22.0345), the binding energy (− 16.5301), and the binding affinity (− 201.4108) for BvrR protein. DrugBank ID (DB00569) had the docking score (− 28.3066), the binding energy (− 18.9236), and the binding affinity (− 267.4938) for OMP25. Also, for the OMP31 protein, DrugBank ID (DB01111) owned the docking score (− 24.7229), the binding energy (− 18.2337), and the binding affinity (− 174.4778). In particular, the docked complex of the three target proteins (BvrR, OMP25 and OMP31) with the top five hits of each of them: first from ZINC (ZID 022035210, ZID 003954125, ZID 019366239, ZID 0169129, and ZID 00168942), (ZID 02,756,403, ZID 04086383, ZID 09,172,386, ZID 00172013, and ZID 00602468), and (ZID 22551309, ZID 00154214, ZID 10847947, ZID 00463723, and ZID 04854502) (Figs. 4, 5 and 6) and second from DrugBank (DB01901, DB01999, DB01141, DB01741, and DB01753), (DB00569, DB01901, DB01141, DB02516, and DB00362) and (DB00569, DB01111, DB02524, DB00006, and DB01141) (Supplementary Figs. 1–3).

**Table 19:** Molecular docking of BvrR protein using MOE software integrated with ZINC database and ADMElab-2 server.

Compound	mseq	ZINC database ID	Docking score (S)	Binding affinity Kcal/Mol	Binding energy Kcal/Mol	Drug like properties							
						MW	Hydrogen bond acceptors	Hydrogen bond donor	Topological polar surface area (Å2) (TPSA)	No. of Rotatable Bonds (nRot)	logS	logD	logP
**1**	**1829**	**022035210**	** − 16.8744**	** − 58.3099**	** − 13.1624**	**245.21**	**5**	**2**	**47.97**	**8**	**0.496**	** − 0.525**	** − 0.859**
**2**	**2009**	**003954125**	** − 15.5383**	** − 84.7323**	** − 12.6866**	**246.04**	**8**	**4**	**149.2**	**4**	** − 0.915**	**4.76**	**1.352**
**3**	**9132**	**019366239**	** − 15.0681**	** − 61.8955**	** − 13.0549**	**204.11**	**6**	**4**	**98.66**	**7**	** − 0.79**	** − 0.661**	** − 3.26**
**4**	**12,856**	**01695129**	** − 15.1195**	** − 78.1743**	** − 13.5454**	**217.97**	**2**	**0**	**26.3**	**4**	** − 2.408**	**2.65**	**3.271**
**5**	**12,860**	**00168942**	** − 15.1922**	** − 72.4002**	** − 13.1458**	**280**	**5**	**0**	**73.06**	**3**	** − 3.488**	**2.461**	**2.176**
6	14,006	001737892	− 15.1030	− 73.0109	− 13.7932	245.17	6	6	118.44	9	− 0.637	− 0.677	− 2.862
7	9	00408917	− 14.0722	− 64.4047	− 13.8712	321.1	5	1	68.63	3	− 5.527	3.385	4.555
8	1769	091723860	− 14.1525	− 70.9786	− 11.9068	237.16	6	3	80.06	3	− 0.407	− 0.509	− 1.188
9	1866	020270251	− 14.2118	− 72.7570	− 11.1898	213.18	4	1	27.74	4	0.322	0.181	− 0.574
10	5632	07080053	− 14.3759	− 61.5902	− 12.2912	319.09	5	1	68.54	8	− 4.267	2.663	3.023
11	8073	02348022	− 14.2576	− 67.8386	− 14.0710	395.23	7	0	67.52	3	− 4.29	3.362	4.326
12	8812	40943553	− 14.4593	− 58.2046	− 12.5358	427.02	5	1	56.79	8	− 6.506	3.7	4.739
13	9051	005274090	− 14.1097	− 68.4637	− 14.0478	246.12	7	5	129.72	8	− 0.804	0.115	− 2.621
14	9065	07175417	− 14.6310	− 93.4404	− 14.3095	403.12	6	1	73.86	10	− 5.982	3.75	4.32
15	9395	22435215	− 14.1051	− 97.8297	− 12.8480	449.18	7	1	78.95	9	− 4.081	2.834	2.817
16	9434	003040197	− 14.3342	− 62.9283	− 11.6448	232.03	6	2	95.55	4	− 0.524	− 1.426	− 2.011
17	10,148	21305182	− 14.3352	− 55.2878	− 14.2352	300.2	5	3	72.94	7	− 1.538	1.542	1.139
18	14,385	00172116	− 14.8699	− 88.2810	− 14.8585	311.03	4	0	43.85	3	− 4.376	3.424	3.454
19	15,390	00497800	− 14.2955	− 55.4569	− 14.1880	314.02	4	2	65.98	1	− 4.598	3.083	3.74
20	15,650	74065906	− 14.5277	− 71.8478	− 12.5528	299.16	4	1	55.4	6	− 3.724	3.258	3.171

Significant values are in** bold**.

**Table 20:** Molecular docking of OMP25 protein using MOE software integrated with ZINC database and ADMElab-2 server.

Compound	mseq	ZINC database ID	Docking score (S)	Binding affinity Kcal/Mol	Binding energy Kcal/Mol	Drug like properties							
						MW	Hydrogen bond acceptors	Hydrogen bond donor	Topological polar surface area (Å2)(TPSA)	No. of Rotatable Bonds (nRot)	logS	logD	logP
**1**	**1823**	**02756403**	** − 16.0424**	** − 61.8023**	** − 14.1846**	**430.06**	**5**	**2**	**58.95**	**6**	** − 6.152**	**3.895**	**4.895**
**2**	**1430**	**04086383**	** − 15.4545**	** − 80.6489**	** − 12.3045**	**332.08**	**3**	**2**	**41.13**	**7**	** − 6.154**	**4.521**	**4.693**
**3**	**1769**	**09172386**	** − 15.7381**	** − 66.3356**	** − 12.5221**	**400.07**	**4**	**1**	**51.22**	**8**	** − 4.475**	**3.611**	**4.318**
**4**	**6642**	**00172013**	** − 15.1870**	** − 68.2207**	** − 14.3979**	**273.09**	**4**	**3**	**45.32**	**6**	** − 4.025**	**3.492**	**3.017**
**5**	**845**	**00602468**	** − 14.1645**	** − 88.3150**	** − 13.0884**	**420.14**	**4**	**1**	**55.76**	**9**	** − 5.027**	**4.042**	**4.7**
6	1248	06385634	− 14.3651	− 75.6116	− 13.4581	269.14	3	0	29.54	6	− 3.151	3.263	3.33
7	1376	00144608	− 14.4405	− 69.2788	− 13.8851	308.05	3	1	24.5	2	− 6.628	3.934	4.912
8	1695	06774077	− 14.2986	− 61.8526	− 13.4795	301.1	4	0	42.16	4	− 6.661	4.171	5.182
9	1756	09022581	− 14.3340	− 68.3018	− 12.3861	439.09	7	0	89.98	9	− 4.207	2.217	2.079
10	1767	09162570	− 14.3654	− 66.0381	− 14.4081	441.14	8	2	100.99	11	− 4.626	2.365	2.958
11	1779	70821007	− 14.3297	− 64.1927	− 13.4248	256.02	5	4	83.8	4	− 2.613	2.008	1.764
12	1784	01936510	− 14.5485	− 60.1780	− 13.9095	520.97	6	1	81.7	10	− 6.241	3.565	5.663
13	1818	03679565	− 14.1939	− 74.5382	− 13.4694	388.03	6	0	73.81	6	− 5.846	3.659	4.67
14	1842	23801148	− 14.3834	− 65.4343	− 14.2173	139.11	3	2	46.33	1	− 0.127	0.65	− 1.511
15	1859	08440448	− 14.3806	− 64.4445	− 13.1837	139.11	3	2	46.33	1	− 0.127	0.65	− 1.511
16	1864	02286212	− 14.5748	− 61.0770	− 14.1747	387.19	9	2	109.04	5	− 2.361	0.78	0.759
17	1910	02011239	− 14.4531	− 71.4605	− 13.4548	376.25	3	1	26.71	3	− 4.9	4.276	4.652
18	2003	00203409	− 14.1781	− 76.6683	− 12.5920	333.08	5	2	75.63	7	− 3.255	2.267	3.033
19	2016	00857577	− 14.3100	− 77.4464	− 12.6678	380.09	5	2	59.59	5	− 7.023	3.846	5.156
20	2801	03438390	− 14.5130	− 62.2155	− 15.7829	257.11	4	1	55.4	6	− 3.265	2.383	2.447

Significant values are in **bold**.

**Table 21:** Molecular docking of OMP31 protein using MOE software integrated with ZINC database and ADMElab-2 server.

Compound	mseq	ZINC database ID	Docking score (S )	Binding affinity Kcal/Mol	Binding energy Kcal/Mol	Drug like properties							
						MW	Hydrogen bond acceptors	Hydrogen bond donor	Topological polar surface area (Å2)(TPSA)	No. of rotatable bonds (nRot)	logS	logD	logP
**1**	**2339**	**22551309**	** − 14.7566**	** − 57.0108**	** − 11.3077**	**436.05**	**5**	**1**	**66.48**	**7**	** − 4.317**	**3.077**	**3.526**
**2**	**55**	**00154214**	** − 13.3158**	** − 67.0866**	** − 11.7225**	**331.12**	**8**	**3**	**113.73**	**9**	** − 4.364**	**2.58**	**2.432**
**3**	**118**	**10847947**	** − 13.4718**	** − 66.4300**	** − 13.2362**	**413.08**	**7**	**2**	**98.66**	**6**	** − 4.38**	**2.749**	**2.285**
**4**	**1**	**00463723**	** − 13.2875**	** − 79.1861**	** − 9.8985**	**286.17**	**4**	**1**	**49.41**	**9**	** − 3.032**	**2.621**	**2.762**
**5**	**215**	**04854502**	** − 13.3222**	** − 89.6962**	** − 10.6307**	**357.21**	**6**	**1**	**55.73**	**6**	** − 4.369**	**3.356**	**2.916**
6	498	00209757	− 13.2673	− 67.5074	− 11.6586	232.1	2	0	28.72	2	− 4.925	3.577	3.629
7	549	01997915	− 13.1373	− 68.6614	− 10.3566	300.21	2	2	40.46	1	− 4.06	4.526	4.621
8	3314	00249136	− 13.0843	− 77.5276	− 11.5416	281.04	2	1	29.1	3	− 3.899	3.454	3.821
9	2330	08406377	− 13.3601	− 71.4889	− 10.9435	382.15	6	1	65.01	4	− 5.409	2.374	4.335
10	2382	04249312	− 13.3235	− 65.1034	− 10.9282	382.18	9	2	108.15	4	− 2.817	1.537	1.253
11	2544	00213986	− 13.2476	− 55.7252	− 11.8620	315.1	9	1	110.37	3	− 2.359	1.035	0.733
12	2606	01185059	− 13.8139	− 71.8234	− 12.6702	352.96	2	1	29.1	4	− 6.712	3.493	4.87
13	2671	03763174	− 13.0252	− 55.6920	− 10.9952	487.14	6	1	68.23	8	− 4.161	3.796	4.338
14	4621	04945141	− 13.2982	− 62.6498	− 9.7842	411.19	9	0	83.52	5	− 4.282	3.137	2.848
15	2976	06291344	− 13.0929	− 77.4419	− 10.8202	358.19	6	1	67.87	7	− 4.898	2.706	3.352
16	3029	23007714	− 13.0467	− 59.5558	− 11.7799	391.15	6	0	69.63	5	− 5.156	4.033	4.148
17	3101	00365021	− 13.1618	− 77.2706	− 10.5110	281.14	3	1	38.33	4	− 5.046	3.534	3.911
18	5803	06540519	− 13.0573	− 66.0322	− 10.7564	320.14	7	3	98.72	7	− 1.952	1.589	1.386
19	7074	00458067	− 13.6052	− 64.8078	− 13.1387	319	3	0	29.54	6	− 3.675	3.006	3.273
20	7128	00156973	− 13.0802	− 67.6662	− 11.2834	129.08	3	2	49.33	1	0.025	− 0.678	− 2.836

Significant values are in **bold**.

#### Toxicity prediction

**Figure 7:**
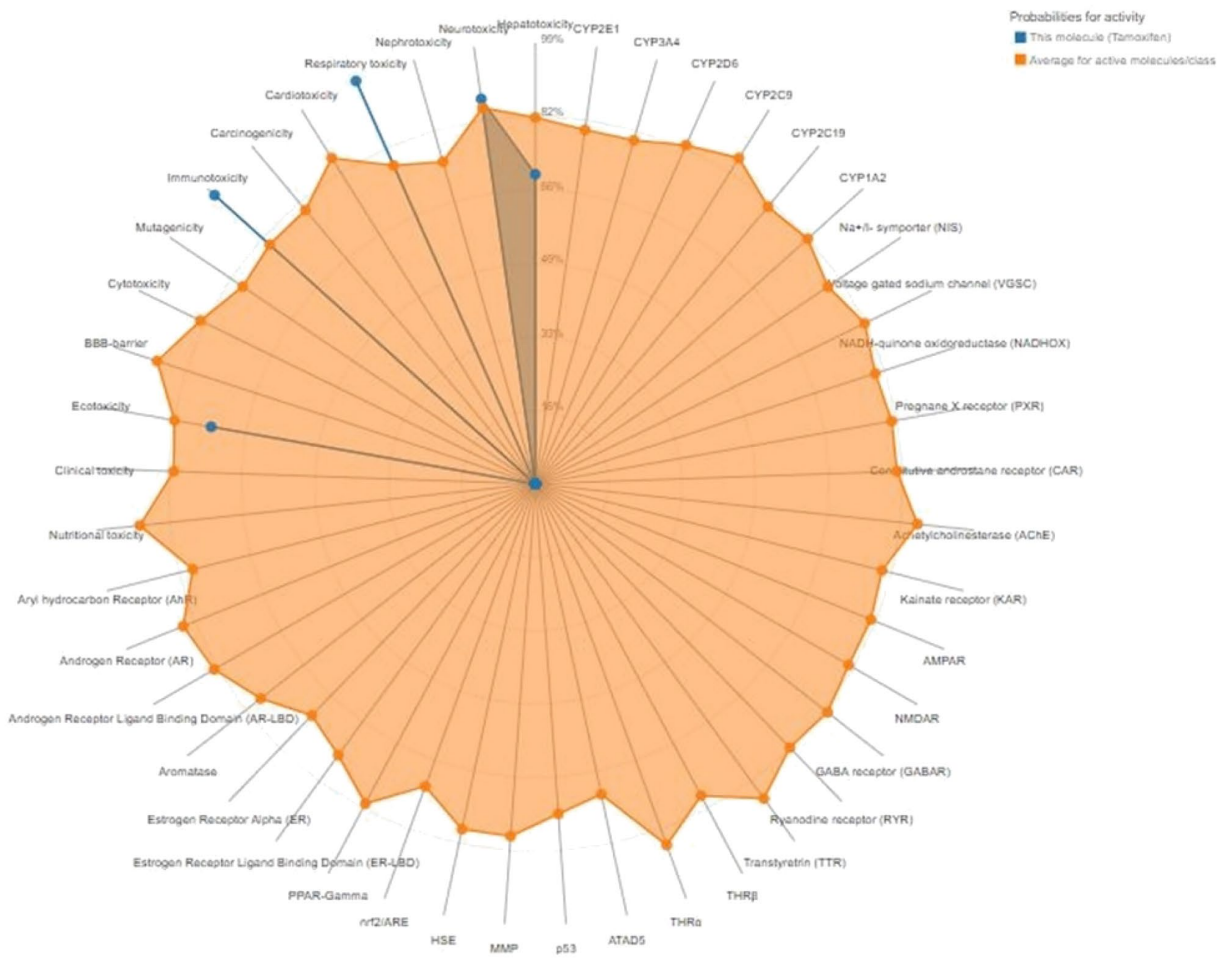
Toxicity prediction of the lead compound using ProTox II server.

The ProTox II server was used to predict the top five lead compounds from the ZINC database for each protein. As shown in (Table 22), these compounds had almost the same probability. Furthermore, they exhibited no toxicity with nephrotoxicity, cardiotoxicity, carcinogenicity, mutagenicity, cytotoxicity, blood–brain barrier (BBB) penetration, clinical toxicity, or nutritional toxicity, as shown in (Fig. 7).

**Table 22:** Toxicity prediction of the best lead compounds of each protein using ProTox II server.

Protein	Target	Prediction	Probability
Three target proteins (BvrR, OMP25, and OMP31)	Nephrotoxicity	Inactive	0.90
	Cardiotoxicity	Inactive	0.77
	Carcinogenicity	Inactive	0.62
	Mutagenicity	Inactive	0.97
	Cytotoxicity	Inactive	0.93
	BBB-barrier	Inactive	1
	Clinical toxicity	Inactive	0.56
	Nutritional toxicity	Inactive	0.74

#### Binding interactions of finally selected compounds

In the docking studies, it was observed that all the finally selected hits showed significant binding interactions with the important residues of the target proteins. The previously listed 15 compounds from the ZINC database, which demonstrated a strong binding affinity, lower binding energy, and good docking score, were observed and showed the binding interaction with the three target proteins binding site residues.

**Figure 8:**
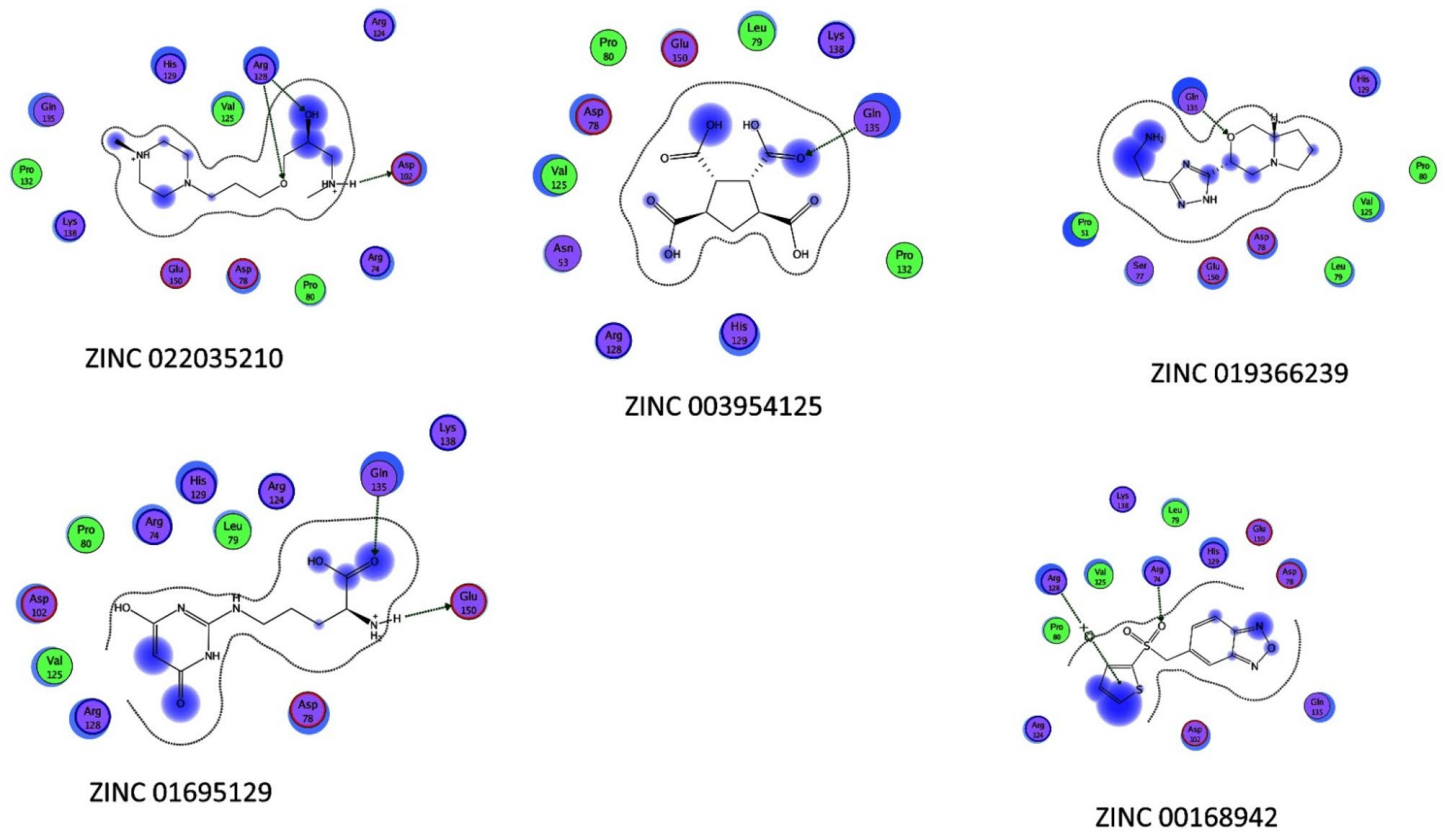
Ligand interaction of BvrR protein and molecules from ZINC database.

For the BvrR target protein, Gln 135 residue made a polar interaction with the (ZINC 019366239) compound and acted as a donor. Also, (the ZINC 022035210) compound established interactions with Arg 128 residue, which had two donor interactions, and Asp 102 residue which performed as a hydrogen acceptor from NH + . Gln 135 residue of docking pose of (ZINC 003954125) compound display as a donor. In (ZINC 01695129) compound, it made polar and acidic interactions with Glu 150 and Gln 135 residues, which behaved as hydrogen acceptors and as donors, respectively. What’s more, Arg 74 and 128 were involved in basic interactions with the (ZINC 00168942) compound. They exhibited as donors and made arene-cation interactions with benzene rings, respectively (Fig. 8).

**Figure 9:**
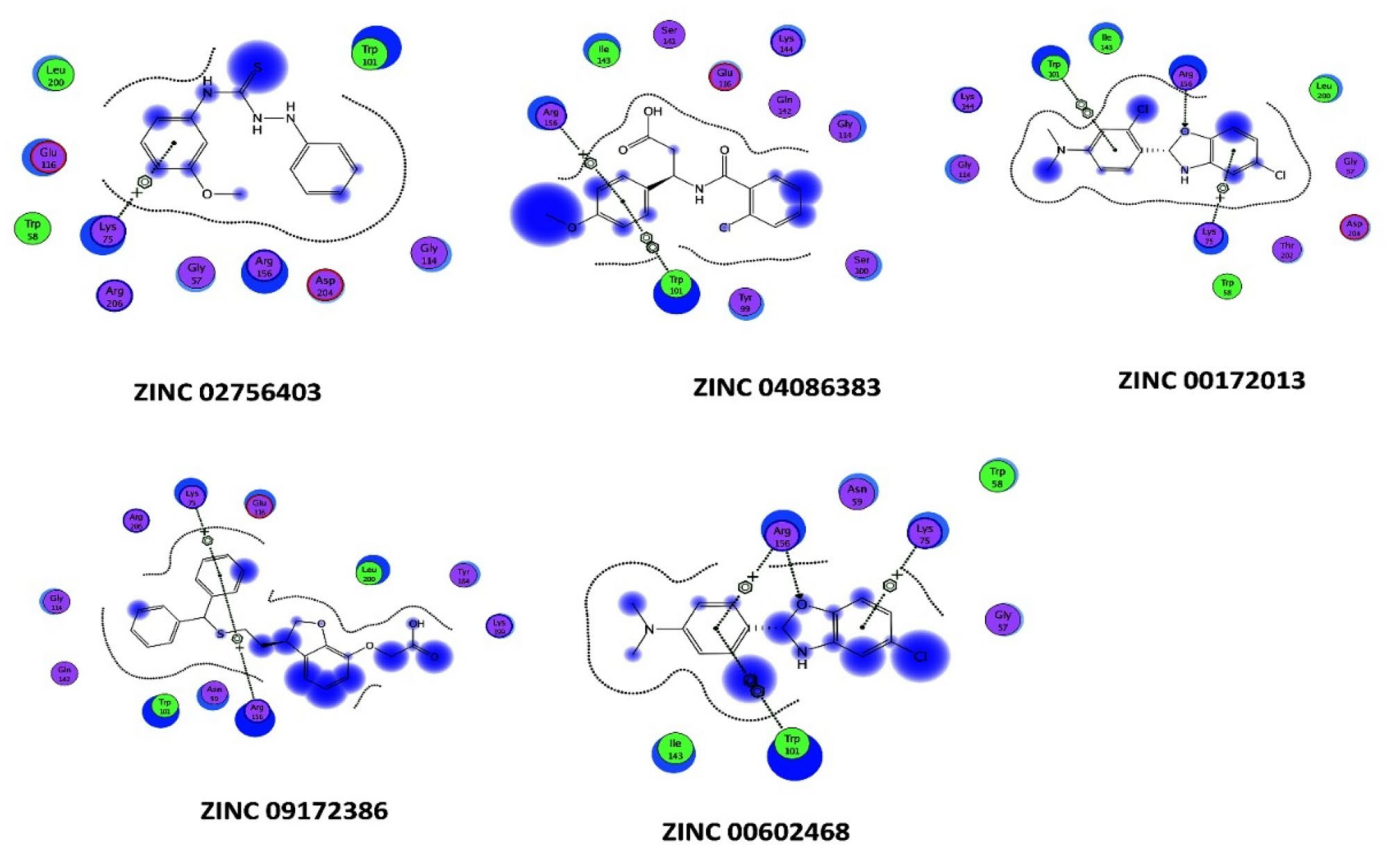
Ligand interaction of OMP25 protein and molecules from the ZINC database.

For the OMP25 target protein, Lys 75 residue had basic interaction and made an arene-cation with the benzene ring of the (ZINC 02756403) compound. While Trp 101 and Arg 156 residues were involved in greasy and basic interactions, respectively in the (ZINC 04086383) compound and do arene-cation interaction with the benzene ring of the compound. According to the docking pose of the (ZINC 00172013) compound, basic interactions with Lys 75 and Arg 156 residues occurred. They made arene-cation with the benzene ring of the ligand and acted as donors for carbonyl oxygen, respectively. Also, Trp 101 has a greasy interaction and activates arene-arene with the benzene ring of the ligand. Moreover, Lys 75 and Arg 156 residues were involved in different basic interactions with the (ZINC 09172386) compound and made arene-cation with its benzene ring. Furthermore, Arg 156 residue interacted with the (ZINC 00602468) compound and had two interactions; it operated as a donor for carbonyl oxygen and performed arene-cation with the benzene ring of the compound (the same as what Lys 75 residue made). Additionally, Trp 101 created an arene-arene interaction benzene ring with the ligand (Fig. 9).

**Figure 10:**
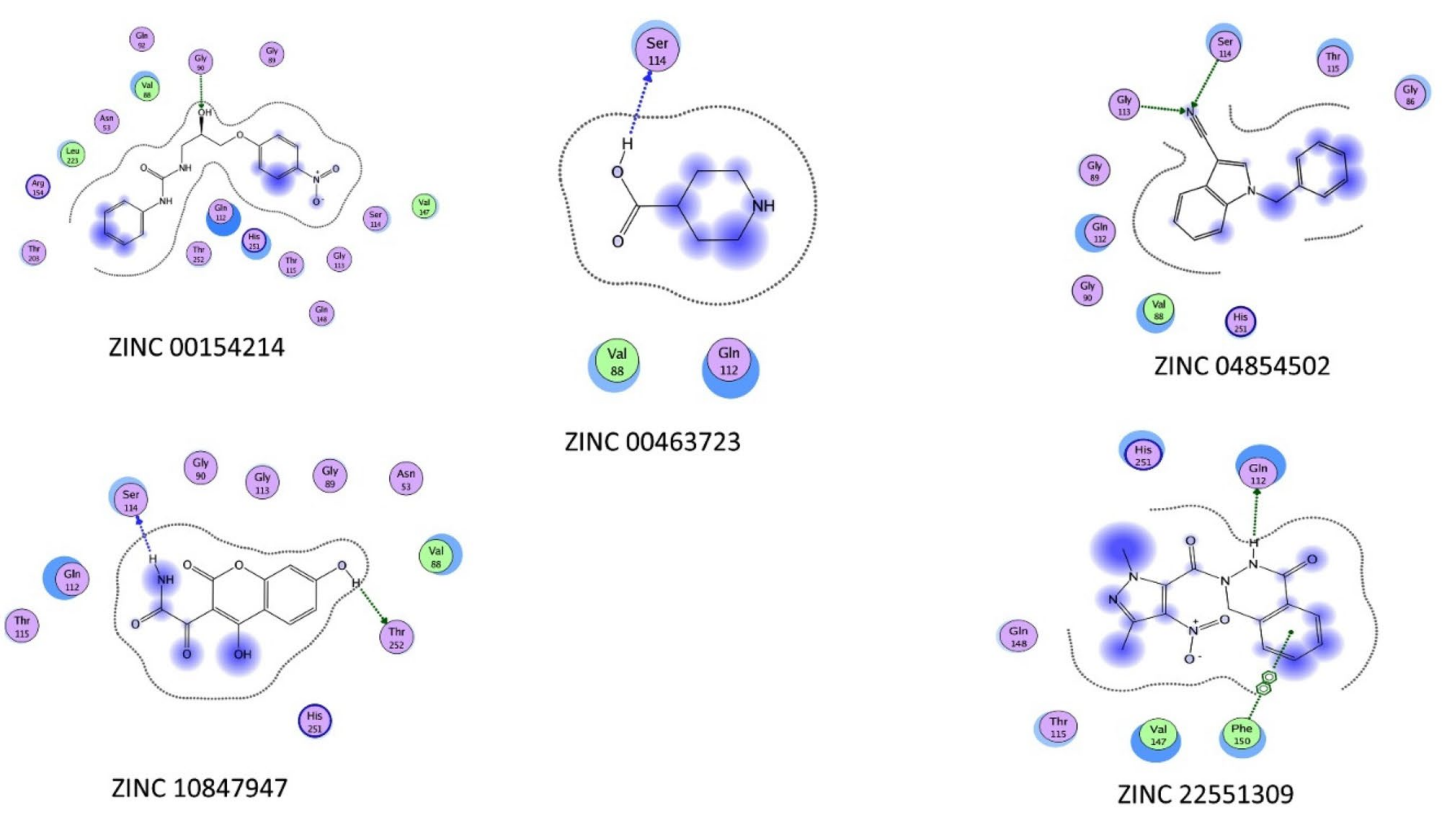
Ligand interaction of OMP31 protein and molecules from the ZINC database.

It was observed that the compound (ZINC 00154214) established an interaction with the Gly 90 residue of the OMP31 target protein. This residue acted as a donor of hydrogen to a hydroxyl group of the compound. While Ser 114 residue interacted with the (ZINC 00463723) compound and displayed as an acceptor of hydrogen. Also, Ser 114 and Gly 113 residues interacted with (ZINC 04854502) compounds and exhibited as donors. Furthermore, (ZINC 10847947) compound made interactions with Ser 114 and Thr 252 residues, which played as hydrogen acceptors from nitrogen monohydride and acceptor, respectively. According to the docking pose of the (ZINC 22551309) compound, Gln 112 interacted with it and operated as an acceptor, and Phe150 made an arene-arene with a benzene ring of it (Fig. 10).

### Molecular dynamic simulation

To predict the nature and the interactions of the docked proteins with their best ligands, a molecular dynamics simulations (MDS) study was applied to BvrR + ZINC 022035210, OMP25 + ZINC 02756403, and OMP31 + ZINC 22551309 complexes. The study used parameters such as root mean square deviations (RMSDs), gyration radius (Rg), root mean square fluctuations (RMSFs), and solvent-accessible surface area (SASA).

**Figure 11:**
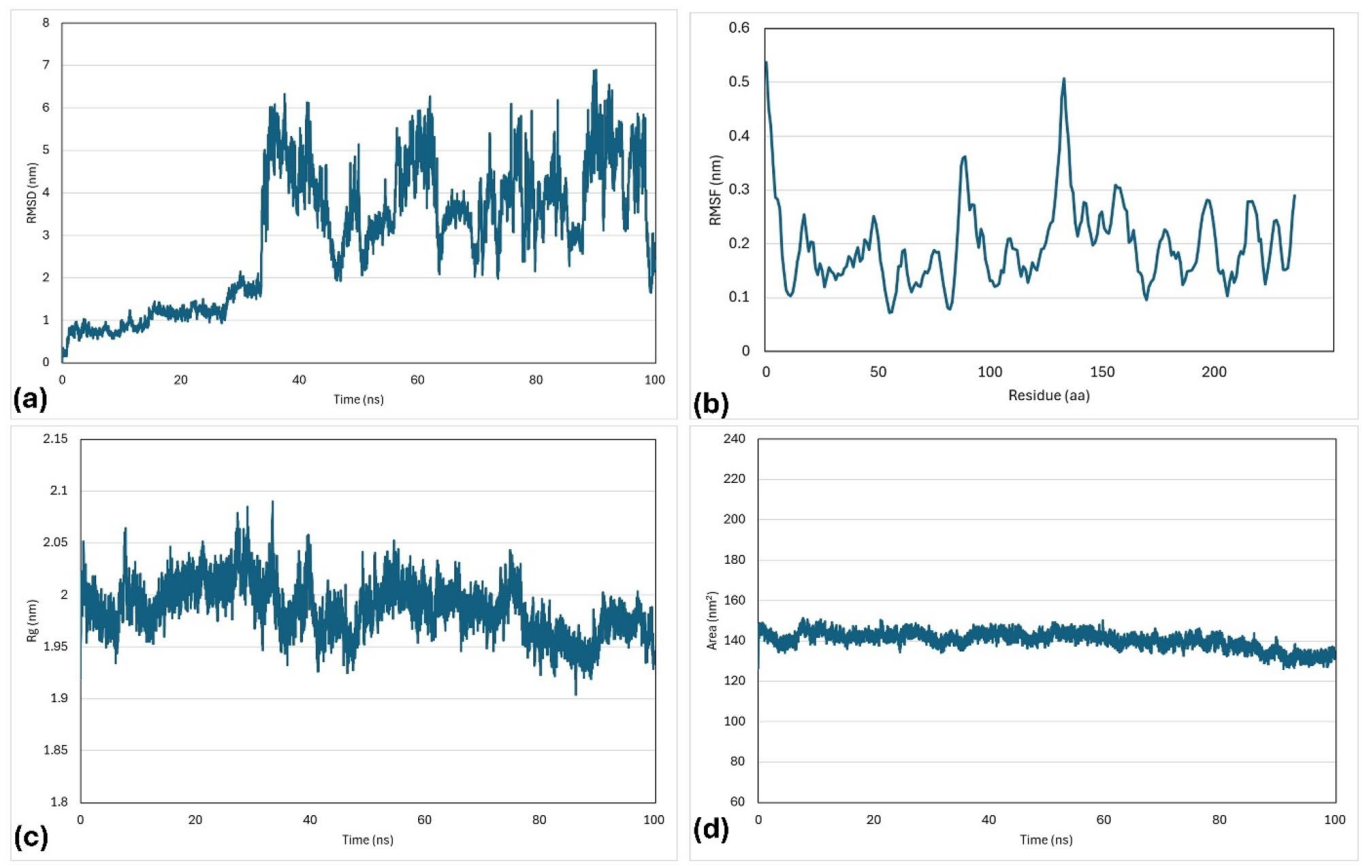
Molecular dynamic simulation of the docked BvrR protein and ZINC 022035210 complex generated at 100 ns. (**a**) RMSD, (**b**) RMSF, (**c**) Rg, and (**d**) SASA.

The process had a time function of 100 ns, and the changes in bonds and trajectories were analyzed. The RMSD values of the BvrR protein and its ligand were initially observed to increase gradually from 1 to 2 nm. This trend continued until 38 ns of simulation had elapsed, at which point they began fluctuating widely. Overall, the RMSD values ranged from 3 to 6 nm until the end of the simulations. The average RMSF value was 0.20 ± 0.08 nm, with greater fluctuations observed in residues 130 to 140 and 1 to 10. The average Rg was 1.99 ± 0.02 nm, with fluctuation decreasing from 2.05 nm to 1.95 nm. Similarly, the SASA had an average of 140.45 ± 4.23 nm^2^ and remained stable until 80 ns, after which it decreased to 130 nm^2^ (Fig. 11).

**Figure 12:**
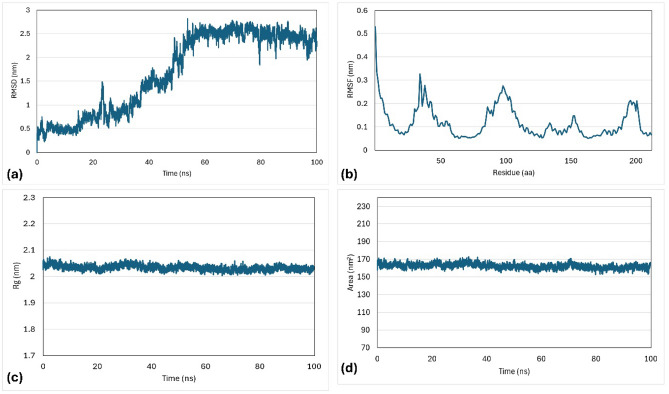
Molecular dynamic simulation of the docked OMP25 protein and ZINC 02756403 complex generated at 100 ns. (**a**) RMSD, (**b**) RMSF, (**c**) Rg, and (**d**) SASA.

The OMP25 and its ligand (ZINC 02756403) were initially observed to increase gradually from 0.4 to 2.4 nm. This trend continued until 50 ns of simulation, then stable region of 2.5 nm during the simulation period of 60 to 100 ns (Fig. 12).

**Figure 13:**
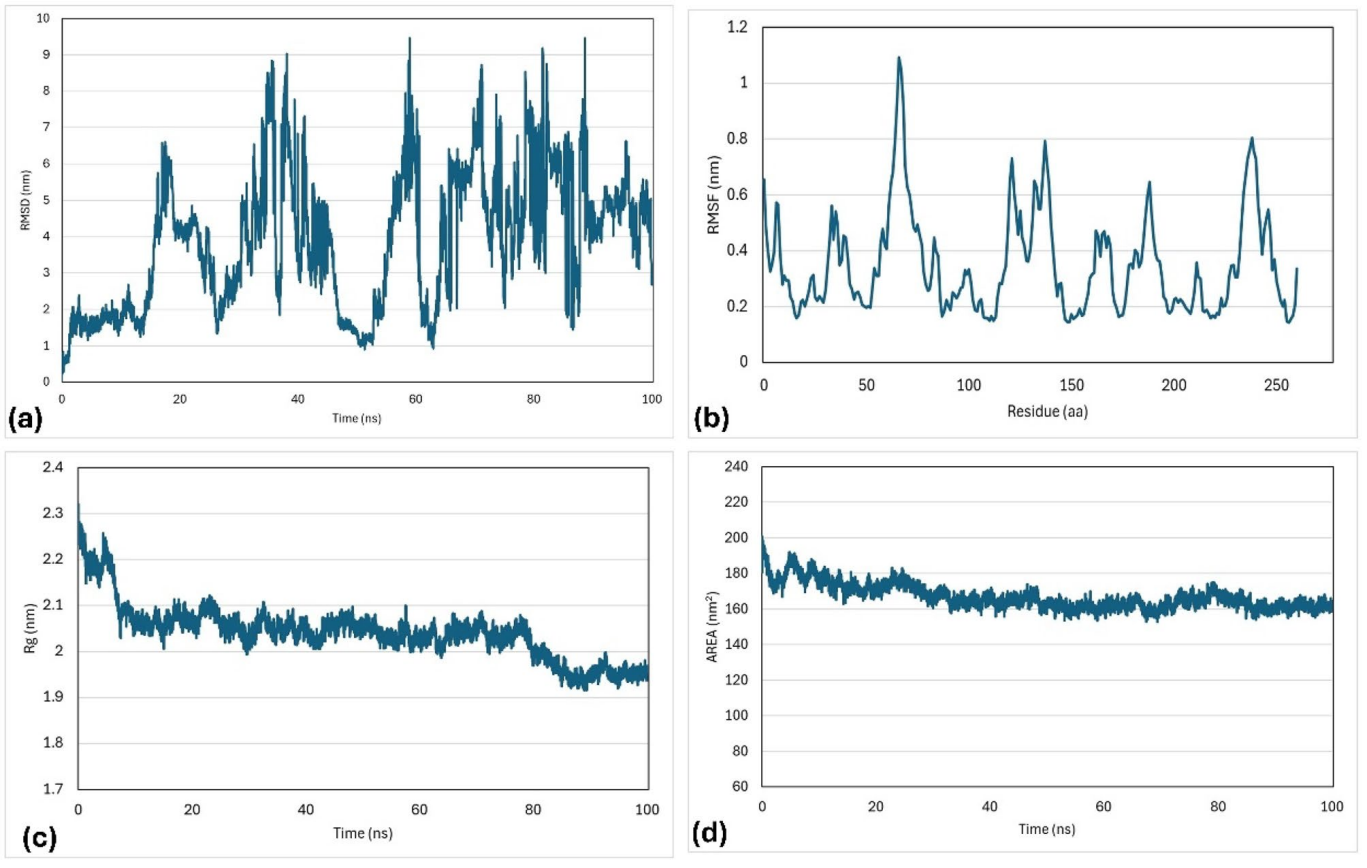
Molecular dynamic simulation of the docked OMP31 protein and ZINC 22551309 complex generated at 100 ns. (**a**) RMSD, (**b**) RMSF, (**c**) Rg, and (**d**) SASA.

The OMP31 and its ligand (ZINC 22551309) showed highly fluctuating RMSD values with a small stable region of 1.7 nm during the simulation period of 3 to 18 ns. The RMSF showed an average of 0.35 ± 0.18 nm, and the highest fluctuating area was between 50 to 80 amino acids. The average Rg value was 2.04 ± 0.06 nm, with stability at 2.05 nm within the range of 10 to 80 ns and 1.95 nm within the range of 85 to 100 ns, supporting the stability of the SASA values throughout the simulation period, with an average of 166.96 ± 7.14 nm^2^ (Fig. 13).

## Discussion

Brucellosis is the most prevalent zoonotic disease globally, causing immense economic losses^20^. Its pathogenicity is due to its virulence factors that facilitate intracellular replication and survival adaptation^21^. This leads to over 500,000 new infection cases annually^22^. According to prior studies, the BvrR/BvrS system is a master regulator of the homeostasis of the cell membrane, nitrogen, and carbon metabolism, and virulence proteins such as OMPs^4^. The BvrR protein identified one domain using the CDD server which is OmpR. It is defined as a DNA-binding response regulator and belongs to the OmpR family that contains winged helix-turn-helix (wHTH) and REC domains. Histidine kinases (HK) and a response regulator protein (RR) are two common components used in the majority of bacterial signal-transduction systems^23^. Our result was agreed with Ref.^24^ who identified that the BvrR protein has sequence that responsible for regulation of the OMP25 protein. The regulatory domain phosphorylation generates a conformational change that leads to the activation of a related domain, which influences the response^25^. The phosphorylable aspartate residue is located in the conserved acidic pocket that serves as the active site^23^. The OmpR/PhoB subfamily is the largest subfamily of RRs and harbors several transcriptional regulators, including the Trans_reg_C domain located at the BvrR protein's C-terminal. This domain is typically accompanied by the REC domain to perform a DNA binding function^26^. Because of these domain structures, the BvrR protein is considered the transductor of environmental signals and adaptation of the intracellular lifestyle of *Brucella* and this is the same finding of Ref.^4,24^.

Additionally, OMP25 and OMP31 proteins were stable, very conservative, difficult to degrade, and triggered potent immune response^27^. These two proteins were identified with the same domain, LomR, using the CDD server and this agreed with Ref.^28^ who discovered the conserved candidate antigens among three *Brucella* species. It is described as opacity proteins and associated surface antigens [membrane/envelope biosynthesis/Cell wall]^29^. The Opacity-associated (Opa) adhesin proteins are the main phase-variable proteins present in Pathogenic *Neisseria* species at the outer membrane, enabling them to evade the human immune system for extended periods, just like *Brucella*. These proteins have a role in bacterial adhesion, entrance, and interaction with the host immune system inhibition in T, and B cells and innate inflammatory reactions^30^. Furthermore, these proteins are linked to other porins, which act as large aqueous channels permitting the entry and selection of hydrophilic substances through a molecular sieve in the gram-negative bacteria cell wall^31^.

Therefore, it was significant to select these three target proteins (BvrR, OMP25, and OMP31) as candidate proteins for constructing a multi-epitope vaccine against *Brucella* due to their important structural function. As far as we are aware, none of these three candidate proteins have been used to design multi-epitope vaccines based on them. Hence, this study gave comprehensive insights into the initial stages of vaccine development, including the secondary and tertiary structure of proteins, and cell epitopes. The secondary and tertiary structures of these three target proteins are analyzed to obtain the allocation and the percentage of alpha-helix, beta-sheet, random coil, and beta-turn in the protein structure. The random coil and beta-turn regions, primarily on the protein surface, are crucial for forming linear B cell epitope regions^32^, while the alpha-helix and beta-sheet are necessary for preserving the protein's secondary structure integrity^33^. The prediction of the secondary structure in this study using the PredictProtein server agreed with a study conducted by Ref.^34^. This study combines immunoinformatics and virtual screening techniques to identify potential epitopes for a designed multi-epitope vaccine against brucellosis and potential drug candidates for inhibiting the BvrR, OMP25, and OMP31 proteins in *Brucella*.

A posttranslational modification (PTM) detects the overall protein structure and function in several biological processes including replication, transcription, translation, apoptosis, cell signaling, and others^35^. Protein phosphorylation is considered one of the most common and vital PTMs, as various enzymes and receptors are activated or deactivated through phosphorylation or dephosphorylation actions, via kinases as well as phosphatases^36^. Casein kinase II (CK-2), is an active Ser/Thr protein kinase that phosphorylates many numbers of substrates, regulates several signaling pathways, and is associated with a wide range of human diseases, especially tumors^37^. Myristoylation is the lipid modification process of the protein, which is important in protein–protein interactions, cell signaling, and targeting proteins to the plasma membrane and endomembrane systems^38^. Protein glycosylation is deemed to be one of the most significant PTMs, and more than half of all proteins in nature are anticipated to be glycosylated. Putative N-glycosylation locations are specific only to the consensus sequence Asn-Xaa-Ser/Thr. However, protein folding plays an integral part in regulating N-glycosylation, and the existence of the consensus tripeptide alone cannot determine whether an asparagine residue is glycosylated^39^.

Research on *Brucella* subunit vaccines has mainly concentrated on protein vaccines, DNA vaccines, and lipopolysaccharide vaccines. With the quick development of bioinformatics, it is now possible to identify the epitopes of diverse antigens using in silico to create a unique vaccination candidate that can trigger desired immune responses^40^. An essential part of the *Brucella* infection's early phases is played by humoral immunity. However, cellular immunity is primarily responsible for the successful immune response when *Brucella* is present inside the cell^41^. Active T cells are crucial for the immunological response to *Brucella*. Most vaccines, however, can only boost B-cell immunity. *Brucella* is successfully exposed to the outside of cells by CD8 + T cells, which also activates additional germicidal processes. Taking into account the requirement for CD4 + T cells to initiate a proper antibody immune response. The epitope prediction, vaccine construction, and validation have been carried out in this study and our result was agreed with Ref.^42–47^ who designed various vaccines against *Brucella*, SARS-CoV-2, and *Staphylococcus aureus*. EAAAK, GPGPG, and AAY linkers were used to connect B-cell, T-cell and helper epitopes. These linkers mitigate the formation of novel epitopes, which is a significant challenge in enhancing recombinant vaccines, while also boosting binding and expression. The EAAAK linker was chosen as an adjuvant linker. The GPGPG aids in triggering the immune response by enhancing the HTL. To prevent the development of new epitopes, the CTL epitopes were connected using AAY^48,49^. The immunogenicity, non-allergenicity, and toxicity of these epitopes were evaluated through the application of immunoinformatic tools, and the effectiveness of such tools was examined in the research conducted by Ref.^50,51^. The constructed vaccine is highly recommended to be a synthetic multi-epitope vaccine in the future, based on B-T cell epitopes for activation of particular B cells, CTL, and THL to produce full coverage protection and long-lasting immune response in vivo.

Virtual screening techniques are currently considered to be effective means for accelerating the drug discovery process. Docking seeks to anticipate the structure of the ligand–protein interaction by investigating the conformational space of the ligands within the protein's binding site^52^. Here, the docking protocol implemented in the MOE program was applied. The ligands were ordered according to the results of the binding free energy calculation using the Generalized-Born Volume Integral/Weighted Surface Area (GBVI/WSA) in the S field, which is the result of the final stage. The GBVI/WSA is a scoring function that determines the ligand's free energy of binding from a specific position. Lower scores corresponded to more favorable poses for all grading criteria. Following docking and scoring, ranked compounds are post-processed by considering factors such as binding scores derived, the accuracy of the generated pose, metabolic liabilities, unwanted chemical moieties, chemical diversity, desired physicochemical qualities, and lead-likeness. After post-processing, a select few compounds are revealed, and these are tested experimentally^53^.

Because drug-like compounds have favorable absorption, distribution, metabolism, excretion, and toxicity (ADMET) properties, drug-likeness is a crucial factor^54^. The drug score, which assesses a compound's overall potential to be a drug, combines drug-likeness, LogP, logS, MW, and toxicity concerns into one important value. The features of each hit ligand were examined for Lipinski's rule of five to determine the druggability of the recovered hits and this agreed with Ref.^42^. According to Lipinski's "rule of five," drug-like molecules require a MW of less than 500 Da, a logP value of less than 5, and hydrogen bond acceptors and donors of less than 10 and 5, respectively; otherwise, they will have poor penetration or absorption^55^. In the present study, a potent inhibitor of *Brucella* BvrR, OMP25, and OMP31 proteins was successfully identified using a SBVS of the ZINC 20 and DrugBank databases. The top five hits of the BvrR, OMP25, and OMP31 proteins from ZINC were (ZID 022035210, ZID 003954125, ZID 019366239, ZID 01695129, and ZID 00168942), (ZID 02756403, ZID 04086383, ZID 09172386, ZID 00172013, and ZID 00602468) and (ZID 22551309, ZID 00154214, ZID 10847947, ZID 00463723 and ZID 04854502), respectively. These compounds could be considered as lead molecules for inhibition of the three target proteins and exhibit favorable ADMET parameters according to the score of Lipinski’s rule. Otherwise, the best ligands from DrugBank (DB01901, DB01999, DB01141, DB01741, and DB01753), (DB00569, DB01901, DB01141, DB02516, and DB00362) and (DB00569, DB01111, DB02524, DB00006, and DB01141) of BvrR, OMP25, and OMP31 proteins, respectively, were not compatible to Lipinski’s rule for estimation the ADMET parameters because of MW more than 500 Da, hydrogen bond acceptor more than ten, and hydrogen bond donor more than five. Toxicity risk alerts are an indication that the drawn structure may be harmful concerning the risk category specified^56^.

Through immunoinformatics and virtual screening, the research focused on the identification of potential epitopes, which enabled the designing of a multi-epitope vaccine against brucellosis and the identification of potential drug candidates for the BvrR, OMP25, and OMP31 proteins in *Brucella*. The implementation of this strategy resulted in leading molecules that satisfied Lipinski's rule of five and also the right ADMET parameters. These findings may play a critical role in vaccine development and anti-*Brucella* drug discovery. However, more research is warranted to confirm the vaccine protein epitopes and lead compounds in vitro and in vivo.

## Conclusion

In conclusion, domain prediction comes first in target structure prediction. Several bioinformatics servers were used in this study to analyze, predict, and identify protein binding motifs related to the biological functions of the three target proteins (BvrR, OMP25, and OMP31). In addition, this approach predicted regions that were conserved within a family, secondary structure, solvent accessibility, tertiary structure, interaction motifs, and PTM sites. The best models of the 3-D structural proteins for the three target proteins were obtained from AlphaFold and I-TASSER servers after refinement and energy minimization according to C-score, TM-score, RMSD, and Z-score. The other aim of this study was to predict and design a multiepitope vaccine and a new potential drug that can be safe, enter the organism, reach its biological target, and elicit the desired effect for brucellosis in animals and humans, respectively. The three target proteins revealed (5, 6, and 5), (3, 4, and 4), and (4, 5, and 4) dominant B cell, CTL, and THL epitopes prediction, respectively. These overlapped epitopes were used to design a chimeric vaccine with 698 aa, that are highly recommended to be used as MEV and manufactural as a synthetic peptide or vector-based candidate vaccine. Additionally, in this present study, a potent inhibitor of *Brucella* BvrR, OMP25, and OMP31 proteins was successfully identified as a prospective drug with novel binding qualities, better mutant potency profile, unique mechanisms of action, increased potency, and pharmacokinetics through SBVS of the ZINC 20 lead database screening collection. Finally, we reach the best five ligands for each protein as the lead compound to inhibit the function of the three target proteins (BvrR, OMP25, and OMP31). The integration of protein modeling and functional analysis of the three target proteins led to detect the active site, which was utilized in the development of vaccine design using epitope prediction and drug design using virtual screening. The confirmation of the hypothesized epitopes will be performed, and the current lead compounds will be enhanced through additional research. Therefore, there is a requirement for more in vitro and in vivo tests so that the safety, efficacy, and toxicity of the vaccine and drug products can be confirmed. The study has pointed out how medicinal remedies may be designed using bioinformatics, virtual screening, and experimental validation.

## Materials and methods

### Data retrieval

We retrieved the three target protein sequences of *Brucella* from the UniProt Knowledgebase (UniProtKB). BvrR protein is 237 amino acids (accession number O67996), OMP25 protein is 213 amino acids (accession number Q44664), and OMP31 protein is 261 amino acids (accession number F8WJS1).

### Domain prediction

The initial step for the 3-D structure prediction is domain separation accurately. The domains of the three proteins (BvrR, OMP25, and OMP31) were predicted using CDD tool^57^ and SMART tool^58^. CDD is a primary sequence annotation tool that discovers new domain families and gives names and attributions for conserved domain architectures. SMART is a comprehensive analysis tool that can predict protein domain architecture, motif, protein classification, and multiple sequence alignment, providing additional information regarding a protein's structural features, evolutionary origin, and function.

### Secondary structural and solvent accessibility prediction

Nine servers were used for secondary structural and solvent accessibility prediction: PSIPRED^59^, NPS@ SOPMA^60^, CFSSP^61^, PSSpre^62^, Lambada Predict Protein^63^, GOR^64^, PredictProtein^65^, PROTEUS2^66^, and RaptorX server. All these servers are established on neural networks in their methods, while PSIPRED applies two feed-forward neural networks and RaptorX relies on a deep learning machine^67^. The prediction from different servers increases the level of accuracy and reliability of the result by deepening the consensus between them. This approach decreases the error occurrence, while the precision of the secondary structural elements and the solvent accessibility value in a protein sequence increase.

### 3D structure prediction

The Robetta server^68^, Swiss-Model server^69^, I-TASSER server^70^, and AlphaFold server^71^ were used for homology modeling prediction. Meta-servers like the LOMETS3 server use fold recognition prediction^72^. For the *ab-initio* structure method, the I-TASSER, Phyre2 servers^73^, C-QUARK server^14^, and CEthreader^74^ were used. Employing multiple servers for the prediction of 3D structures assures the utilization of several techniques and algorithms, this indirectly contributes to the accuracy of the predictions. Additionally, fusing findings from multiple devices takes care of any biases that can be in individual devices’ algorithms. I-TASSER was the best server according to CASP 11, CASP 12, CASP 13, and CASP 14, while AlphaFold was the best one for CASP 15.

### Model refinement

The DeepRefiner^75^, Galaxy WEB^76^, trRossetta^77^, Modrefiner^78^, PREFMD^79^, and ReFOLD^80^ were used. These servers’ function is to simultaneously enhance local and global structural properties of the basic models, bringing them closer to the native state while requiring little computational effort. The refining approach consists of two steps: the first stage optimizes the hydrogen bonding (HB) network, while the second step uses composite physics and knowledge-based force fields to apply atomic-level energy reduction to the optimized model^78^.

### Model evaluation

The two biggest problems in protein structure prediction are sampling and rating structural models. To rank and assess protein structural models, new large-scale model quality assessment (QA) techniques are combined with model clustering. We evaluated the refinement category predictions through different measures (RMSD, TM-score, Z-score, QMEAN, MolProbity score, clash score, overall quality, and Ramachandran Favored (RF)). These measures evaluated complementary aspects of model quality including dihedral angles, distributions of inter-atomic connections, and overall fold. SAVES meta server^81^, PROCHECK server^82^, TM-Score server^83^, TM-align server^83^, QMEAN^84^, Structure Assessment server^19^, and trRossetta^77^ were used for the evaluation of the three target protein structure prediction.

### Functional motif prediction

The primary advantage of motifs is that they can find remote sequence relationships and protein–protein interactions (PPI). There are two methods for obtaining information on motifs. The first method involved using the PROSITE server, which matched regular expressions with a query sequence^85^. The second method involved employing a SMART server that expressed the sequence data from a multiple sequence alignment using probabilistic models after applying HMMs to retain the information. It also exposed intrinsic features like signal sequences, transmembrane helices, compositionally biased regions, and coiled-coil regions, in addition to domains^58^. Also, MotifFinder and Motif Scan tools were used^86^.

### Structural classification

InterPro, SUPERFAMILY 2.0, and CATH databases were used. The InterPro database creates a complete resource for protein classification by combining signatures denoting similar families, domains, or locations with extra data like descriptions, literature references, and GO keywords^87^. Also, the SUPERFAMILY 2.0 database covers superfamily domain annotations for millions of protein sequences that are taken from the NCBI and the UniProtKB^88^. Additionally, the CATH database also recognizes domains in protein structures from the wwPDB and categorizes these into evolutionary superfamilies, providing structural and functional annotations^89^.

### B cell epitope prediction

Prediction of B cell epitope in a more effective way to replace antigens for antibody production. For linear B cell epitope prediction, SVMTriP^90^, Bepipred 2.0 at Immune Epitope Database and Analysis Resource (IEDB), and BCEPS servers were used^50^. Based on sequence properties of the antigen employing amino acid scales and HMMs, all of these servers are regarded as the best in terms of accuracy and area under the curve (AUC)^51^. However, antibodies can recognize discontinuous B-cell epitopes in over 90% of cases. These discontinuous epitopes are made up of sequences that are connected by protein folding characteristics but are separated by long-distance pathogenic protein sequences^91^. The ElliPro server was used to predict 3-D structures using B-cell epitopes^92^.

### T cell epitope prediction

MHC I and MHC II molecules present T cell epitopes, which are recognized by two different T cell subsets, CD8 + and CD4 + T cells, respectively. The human leucocyte antigen (HLA) gene complex is also known as human MHC. After becoming activated, CD4 + T cells recognize HLA-II-restricted antigenic epitopes with lengths of 9 to 22 amino acids and develop into T helper lymphocytes (THL). After being activated, CD8 + T cells recognize HLA-I-restricted 8–12 amino acid residue epitopes and undergo differentiation into cytotoxic T lymphocytes (CTLs). HLA-A * 0101 and HLA-A * 0201 discerned by MHC -I and HLA-DRB * 0701 and HLA-DRB * 0901 discerned by MHC -II were selected in the used servers^41^. RANKPEP^93^, SYFPEITHI^94^, and MHCII-NP^95^ tools were used.

### Prediction of antigenicity, allergenicity, and toxicity

To predict the antigenicity, the VaxiJen v2.0 server was used^96^. Then, the AllergenFP v.1.0 server was applied to calculate the protein’s total sensitivity^97^. In addition, the ToxinPred server was used to predict the toxicity of each selected epitope^98^.

### Vaccine construction and validation

Vaccine construction was done using the PADRE (peptide AKFVAAWTLKAAA) sequence as an adjuvant to bind to the N-terminal of the vaccine. Four main types of linkers such as EAAAK, GPGPG, AAY, and KK were used as described in Ref.^46,44,99^. The final overlapped epitopes from B-cell, CTL, and HLT epitopes were merged to reduce the size of the constructed vaccine. Consequently, the adjuvant, EAAAK, and the linkers of the antigenic epitopes are fused in the order of the adjuvant sequence, CTL, AAY, HTL, GPGPG, B-cell, and KK, to eventually get a consolidated and highly potent vaccine.

After designing the vaccine, the antigenicity, allergenicity, and toxicity of the final constructed vaccine were predicted by Vaxijen v2.0, AllergenFP v.1.0, and ToxinPred servers, respectively. The physicochemical properties also were computed using Expasy's ProtParam^100^.

### Secondary, tertiary structure prediction and refinement of the chimeric vaccine

The secondary prediction of the constructed vaccine was predicted using SOMPA and PredictProtein servers. The tertiary structure prediction was carried out using I-TASSER, AlphaFold, and Swiss Model servers. Then, the 3-D structure of the chimeric vaccine was refined by the Galaxy Refine server^76^. The refined 3-D structure of the vaccine was validated by the structural assessment server, ERRAT, and SAVES meta-server^81^.

### Protein preparation schemes for SBVS

Proper structure-based virtual screening (SBVS) is based on appropriate starting structures for the protein and the ligand. The recommended approach is to first calculate the protonation statuses of the amino acids in the protein and then energy minimization using MOE software version 2009^101^. The default energy minimization parameters were applied, with the forcefield being MMFF94x and a gradient of 0.05, with the "calculate forcefield partial charge" option enabled. The subsequent steps involve assigning hydrogen atoms to improve protein hydrogen bonds and implementing an ideal hydrogen bond network^102^.

### Binding site identification

Executing SBVS frequently also requires binding site detection. The target binding site should ideally be a pocket, typically concave, with several possible hydrogen bond acceptors and donors as well as hydrophobic characteristics. The assignment of hydrogen atoms and improvement of protein hydrogen bonds by an ideal hydrogen bond network is the subsequent step^103^. The MOE program's "Site Finder" feature identified the binding site.

### Compound database preparation for SBVS

The next critical phase of the SBVS process is the creation of compound databases. Small compound databases used in SBVS contain drug-like compounds that are frequently publicly available or may be obtained through purchase or synthesis and have desirable properties, including stability and solubility in aqueous media. ZINC20 database^104^ and DrugBank 5.0 database^105^ were used for virtual screening. 3,687,621 and 11,586 drug lead molecules from the ZINC20 database and DrugBank 5.0 database, respectively were selected and are ready to be screened virtually.

### Molecular docking

Each molecule in the library is virtually docked into the target binding site for each of the three target proteins (BvrR, OMP25, and OMP31) using the MOE program following protein structure preparation^106^. The first scoring function and refinement were adjusted to default London dG and forcefield, respectively. The resulting binding interactions between these hits and protein observed by evaluating binding energy, binding affinity, and docking score were analyzed for binding interactions using LigPlot, which is implemented in MOE^107^.

### ADMET prediction (adsorption, distribution, metabolism, excretion and toxicity)

The docking findings were filtered based on the binding affinity scores and ADME properties since the early assessment of ADME properties can significantly reduce the likelihood of pharmacokinetics-related failure in the clinical phases^108^. In silico screening of the pharmacological properties of ADME was performed and the compounds were evaluated for drug-likeness using the ADMETlab 2.0 web tool^109^. The toxicity of the lead compounds was predicted using ProTox II server^110^. The drug-likeness prediction relied on several rules, including Lipinski, Pfizer, Golden, and GSK. The pharmacokinetics properties observed were molecular weight (MW), H-bond Donor, H-bond Acceptor, No. of Rotatable Bonds (nRot), Topological polar surface area (TPSA), LogS, LogD, and LogP (partition coefficient)^55^.

### Molecular dynamics simulation

GROMACS-2021.3 was utilized to carry out a molecular dynamic simulation of the three target proteins and the best protein–ligand complexes. The interactions were estimated using the CHARMM27 force field, TIP3P water molecule model, and cubic box set at a 10 Å distance from the surrounding edges. Additionally, Na + and Cl– ions at a concentration of 0.15M were applied^111,112^. The electrostatic interactions were evaluated using the particle-Ewald summation^113^, while the estimation of van der Waals (VdW) interactions employed a 10 nm cut-off. The systems were energy-minimized for 5000 steps using the steepest descent and conjugate gradient algorithms followed by NPT and NVT equilibrium phases. Each process lasted 500 ps, and an integration step of 2fs was used^114,115^. Subsequently, MD simulation production runs were conducted for 100 ns, with snapshots taken every 10 ps for analysis purposes^116,117^. The Bibliotheca Alexandrina Supercomputing unit was used for all production runs. Results of the MD simulations were analyzed using the GROMACS-2021.3 package, which included the analysis of protein root mean square deviation (RMSD), root mean square fluctuation (RMSF), radius of gyration (RG), and solvent-accessible surface area (SASA).

### Ethics approval/declarations

This research complies with relevant institutional, national, and international guidelines and legislation. This research does not include any studies with human participants or animals performed by authors.

### Supplementary Information

Supplementary Information.

## Data Availability

All the primary data used in the study were downloaded from UniProt Knowledgebase (UniProtKB) (https://www.uniprot.org/) with accession numbers O67996, Q44664, and F8WJS1. The datasets supporting the conclusions of this article are included within the article (and its additional files).
