# Supplementary material for: Bioinformatics approach for structure modeling, vaccine design, and molecular docking of Brucella candidate proteins BvrR, OMP25, and OMP31
Source: Sci Rep. 2024 May 25;14:11951. doi: 10.1038/s41598-024-61991-7 (PMC11126717; doi:10.1038/s41598-024-61991-7)
Supplement: Supplementary file 1 — Supplementary Information. [file 41598_2024_61991_MOESM1_ESM.pdf]

Bioinformatics Approach for Structure Modeling, Vaccine Design, and Molecular Docking of *Brucella* Candidate Proteins BvrR, OMP25, and OMP31

Alyaa Elrashedy[1*], Mohamed Nayel[1], Akram Salama[1], Mohammed M. Salama[2]& Mohamed E. Hasan[3]

[1]Department of Animal Medicine and Infectious Diseases (Infectious Diseases), Faculty of Veterinary Medicine, University of Sadat City, Egypt.

[2]Physics Department, Medical Biophysics Division, Faculty of Science, Helwan University, Cairo, Egypt

[3]Bioinformatics Department, Genetic Engineering and Biotechnology Research Institute, University of Sadat City, Egypt.

* Corresponding author: alyaaelrshedy96@gmail.com / Alyaa.elrashedy.ms@vet.usc.edu.eg

Table (1) Molecular docking of BvrR protein using MOE software integrated with DrugBank database and ADMElab-2 server

**Compound**	**mseq**	**DrugBank ID**	**Docking score (S)**	**Binding affinity Kcal/Mol**	**Binding energy Kcal/Mol**	**Drug like properties**							
						**MW**	**Hydrogen bond acceptors**	**Hydrogen bond donor**	**Topological Polar Surface Area (Å2)(TPSA)**	**No. of Rotatable Bonds (nRot)**	**logS**	**logD**	**logP**
**1**	**1671**	**DB01901**	**-22.0345**	**-201.4108**	**-16.5301**	**981.77**	**35**	**8**	**536.49**	**21**	**4.277**	**-3.599**	**-6.878**
**2**	**1763**	**DB01999**	**-22.2152**	**-239.0520**	**-21.1477**	**-**	**-**	**-**	**-**	**-**	**-**	**-**	**-**
**3**	**1012**	**DB01141**	**-20.3231**	**-244.9501**	**-14.6593**	**1269.44**	**32**	**17**	**510.14**	**19**	**-1.09**	**0.059**	**-0.489**
**4**	**1525**	**DB01741**	**-19.6677**	**-143.5672**	**-17.7792**	**460.12**	**9**	**5**	**183.61**	**7**	**-3.785**	**-0.336**	**-0.58**
**5**	**1536**	**DB01753**	**-19.4288**	**-170.0049**	**-17.0411**	**760.08**	**25**	**11**	**380.99**	**13**	**-0.183**	**-1.585**	**-4.627**
6	2193	DB02460	-19.1223	-220.7160	-14.6503	880.41	18	8	310.21	18	-2.343	8.307	2.935
7	2245	DB02516	-19.2991	-183.9109	-16.9576	910.21	26	10	386.69	26	-0.363	-1.72	-3.984
8	1157	DB01321	-18.2796	-215.6848	-10.6264	827.47	16	3	206.05	14	-4.423	2.343	2.347
9	1570	DB01792	-18.2082	-176.1484	-14.5449	653.09	22	9	326.15	10	-0.818	-1.355	-3.633
10	2073	DB02332	-18.5194	-215.7934	-16.7288	786.16	24	11	364.55	13	-3.092	-1.157	-3.663
11	2214	DB02483	-18.1426	-145.0591	-18.5015	687.11	21	7	299.48	11	-0.94	-1.798	-2.724
12	2372	DB02654	-18.6293	-190.3194	-16.6950	801.15	25	11	383.16	13	-3.875	-1.229	-3.427
13	2711	DB03020	-18.2017	-171.3336	-19.0673	663.11	21	10	331.35	11	-2.051	-1.589	-3.086
14	571	DB00686	-17.0092	-150.7103	-13.9196	601.92	21	6	322.55	10	2.065	-3.078	-4.03
15	1229	DB01421	-17.1831	-187.0086	-15.0888	615.3	19	18	347.32	9	-0.783	-2.618	-3.892
16	1597	DB01820	-17.2151	-163.4431	-12.1031	533.22	10	4	153.11	15	-3.136	1.132	3.009
17	1971	DB02223	-17.9534	-214.1990	-17.0206	814.28	23	13	390.46	28	-3.446	-0.448	-1.978
18	2079	DB02338	-17.2031	-192.6741	-16.5976	745.09	24	11	364.15	13	-0.523	-1.325	-4.421
19	2228	DB02468	-17.0393	-163.6645	-17.9193	694.22	8	2	92.63	9	-4.047	5.36	6.012
20	2290	DB02563	-17.6831	-209.2480	-15.8694	865.19	24	10	363.63	26	-2.348	-0.647	-1.818

Table (2) Molecular docking of OMP25 protein using MOE software integrated with DrugBank database and ADMElab-2 server

Compound	mseq	DrugBank ID	Docking score (S)	Binding affinity Kcal/Mol	Binding energy Kcal/Mol	Drug like properties							
						MW	Hydrogen bond acceptors	Hydrogen bond donor	Topological Polar Surface Area (Å2)(TPSA)	No. of Rotatable Bonds (nRot)	logS	logD	logP
**1**	**456**	**DB00569**	**-28.3066**	**-267.4938**	**-18.9236**	**1506.95**	**52**	**19**	**805.48**	**30**	**4.704**	**-3.852**	**-8.388**
**2**	**1671**	**DB01901**	**-26.3782**	**-211.6280**	**-20.2794**	**981.77**	**35**	**8**	**536.49**	**21**	**4.277**	**-3.599**	**-6.878**
**3**	**1012**	**DB01141**	**-25.3459**	**-305.5519**	**-13.9942**	**1269.44**	**32**	**17**	**510.14**	**19**	**-1.09**	**0.059**	**-0.489**
**4**	**2245**	**DB02516**	**-25.1994**	**-183.6110**	**-19.9472**	**910.21**	**26**	**10**	**386.69**	**26**	**-0.363**	**-1.72**	**-3.984**
**5**	**255**	**DB00362**	**-24.4539**	**-279.6868**	**-14.0054**	**1139.51**	**24**	**14**	**377.42**	**15**	**-3.63**	**1.443**	**1.154**
6	982	DB01111	-24.2395	-223.7473	-17.9453	1633.59	44	18	706.71	48	-2.043	-5.997	-4.437
7	1006	DB01135	-23.1625	-256.9707	-13.6432	1034.53	18	0	163.36	29	-1.5	4.295	1.961
8	1733	DB01969	-23.8910	-168.4187	-19.6803	877.11	24	10	363.63	24	-1.816	-1.126	-2.919
9	2089	DB02349	-23.9929	-225.7690	-21.3783	809.15	26	14	418.88	16	-0.979	-1.791	-4.961
10	2745	DB03059	-23.7666	-239.4576	-18.0593	851.14	25	10	380.7	24	-1.386	-1.334	-3.685
11	2827	DB03147	-23.0004	-207.6246	-17.8811	785.16	24	10	362.93	13	-2.884	-0.898	-2.557
12	3339	DB03698	-23.6643	-251.4508	-17.7925	994.23	25	11	382.55	32	-3.92	-0.317	-1.447
13	3	DB00014	-22.0249	-197.8759	-9.2489	1268.64	32	20	495.89	43	-2.912	0.272	-0.25
14	8	DB00080	-22.8053	-338.0793	-14.8258	1619.71	43	25	702.02	39	-1.773	-0.242	-1.388
15	407	DB00520	-22.2530	-235.0591	-13.2345	1092.64	25	18	412.03	24	-1.398	0.538	0.015
16	530	DB00644	-22.8118	-262.6792	-10.5054	1181.57	30	19	474.63	40	-3.046	-1.641	-1.429
17	1141	DB01282	-22.4707	-246.9476	-11.9625	987.48	23	13	362.51	21	-2.906	0.494	-0.142
18	2865	DB03186	-22.2630	-166.6394	-18.2952	797.03	27	10	398.98	14	-1.355	-1.455	-4.539
19	3562	DB03933	-22.4460	-258.2363	-18.5955	846.26	16	7	219.42	7	-4.961	1.634	4.433
20	1462	DB01643	-21.3829	-200.8899	-17.6694	322.06	10	4	151.08	4	-0.115	-0.815	-1.599

Table (3) Molecular docking of OMP31 protein using MOE software integrated with DrugBank database and ADMElab-2 server

Compound	mseq	DrugBank ID	Docking score (S)	Binding affinity Kcal/Mol	Binding energy Kcal/Mol	Drug like properties							
						MW	Hydrogen bond acceptors	Hydrogen bond donor	Topological Polar Surface Area (Å2)(TPSA)	No. of Rotatable Bonds (nRot)	logS	logD	logP
**1**	**982**	**DB01111**	**-24.7229**	**-174.4778**	**-18.2337**	**1633.59**	**44**	**18**	**706.71**	**48**	**-2.043**	**-5.997**	**-4.437**
**2**	**456**	**DB00569**	**-23.5798**	**-250.4877**	**-17.5068**	**1506.95**	**52**	**19**	**805.48**	**30**	**4.704**	**-3.852**	**-8.388**
**3**	**2253**	**DB02524**	**-21.5443**	**-181.8506**	**-15.7366**	**717.98**	**27**	**7**	**389.71**	**10**	**-1.22**	**-0.549**	**-1.095**
**4**	**1**	**DB00006**	**-21.9430**	**-205.3993**	**-4.1190**	**2178.99**	**57**	**31**	**901.57**	**86**	**-1.387**	**-1.168**	**-4.722**
**5**	**1012**	**DB01141**	**-21.4048**	**-218.5133**	**-13.1732**	**1269.44**	**32**	**17**	**510.14**	**19**	**-1.09**	**0.059**	**-0.489**
6	2298	DB02572	-20.8548	-131.0261	-12.6487	1793.85	47	18	644.62	64	-3.009	0.095	-0.419
7	8	DB00080	-20.3902	-191.6664	-13.5410	1619.71	43	25	702.02	39	-1.773	-0.242	-1.388
8	4	DB00027	-19.2365	-200.8207	-6.9976	1810.03	35	20	519.89	65	-3.763	7.06	8.818
9	294	DB00403	-19.1467	-165.3161	-10.8859	1351.45	34	19	551.4	47	-1.114	-0.756	-2.304
10	1763	DB01999	-18.5278	-204.4348	-18.0645	-	-	-	-	-	-	-	-
11	684	DB00803	-18.0794	-170.5195	-15.0807	1154.75	29	23	490.66	32	-0.922	-1.471	-1.418
12	2807	DB03126	-18.0047	-143.5828	-13.5726	544.09	16	6	230.47	10	-3.385	-0.568	-1.377
13	3485	DB03853	-17.2976	-155.4261	-17.3660	1114.94	31	12	519.31	14	2.908	-0.146	-2.837
14	2711	DB03020	-17.5493	-160.0864	-15.0235	663.11	21	10	331.35	11	-2.051	-1.589	-3.086
15	2685	DB02992	-17.0050	-139.3520	-13.9686	355.04	12	9	217.24	10	0.014	-2.364	-3.774
16	2600	DB02903	-17.0197	-177.6418	-13.3766	1473.63	41	18	589.24	42	-2.67	0.138	-0.56
17	2370	DB02651	-17.1370	-162.2253	-13.4218	685.1	9	4	145.77	10	-5.675	3.318	4.214
18	2033	DB02290	-17.4593	-142.8897	-13.6089	440.09	15	8	240.33	10	-0.866	-1.392	-2.557
19	1636	DB01861	-17.6873	-143.5749	-14.6638	566.06	19	9	296.99	9	0.353	-2.724	-4.306
20	552	DB00666	-17.7266	-140.5465	-11.5592	1321.64	30	19	472.13	43	-3.821	0.53	0.682

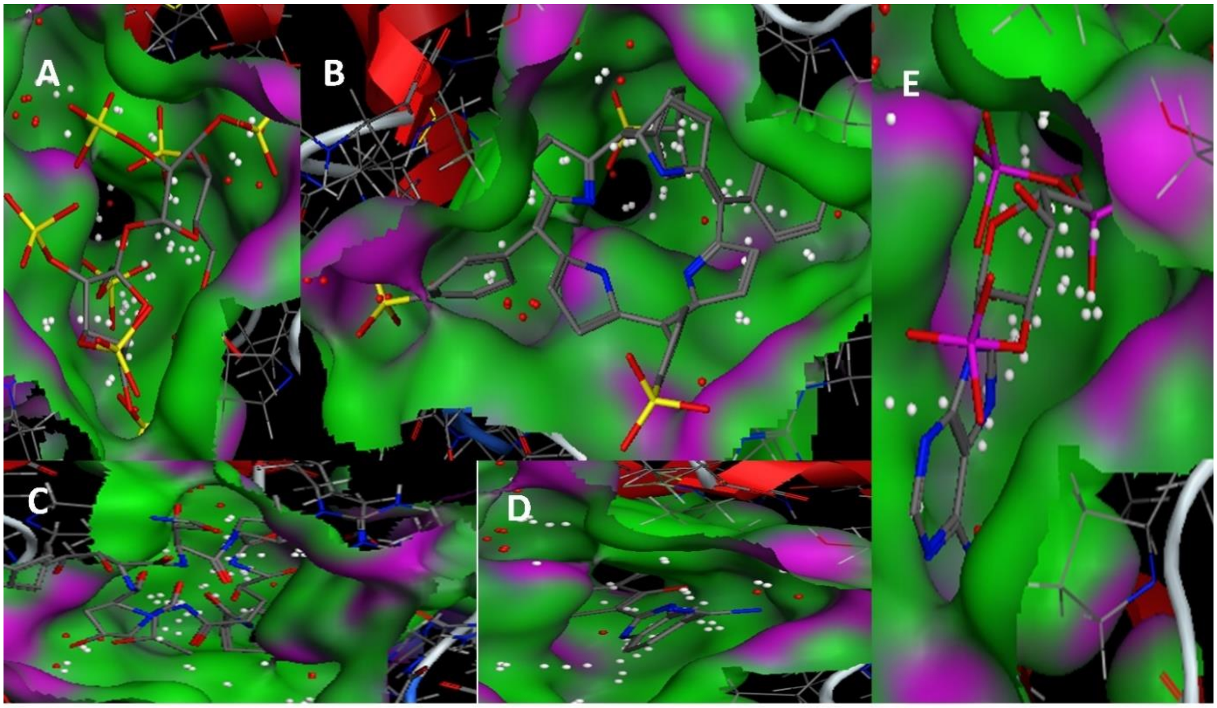

Figure (Supplementary F1): The d docking binding mode of BvrR protein and Drugbank database. (A) DB01901, (B) DB01999, (C) DB01141, (D) DB01741, and (E) DB01753

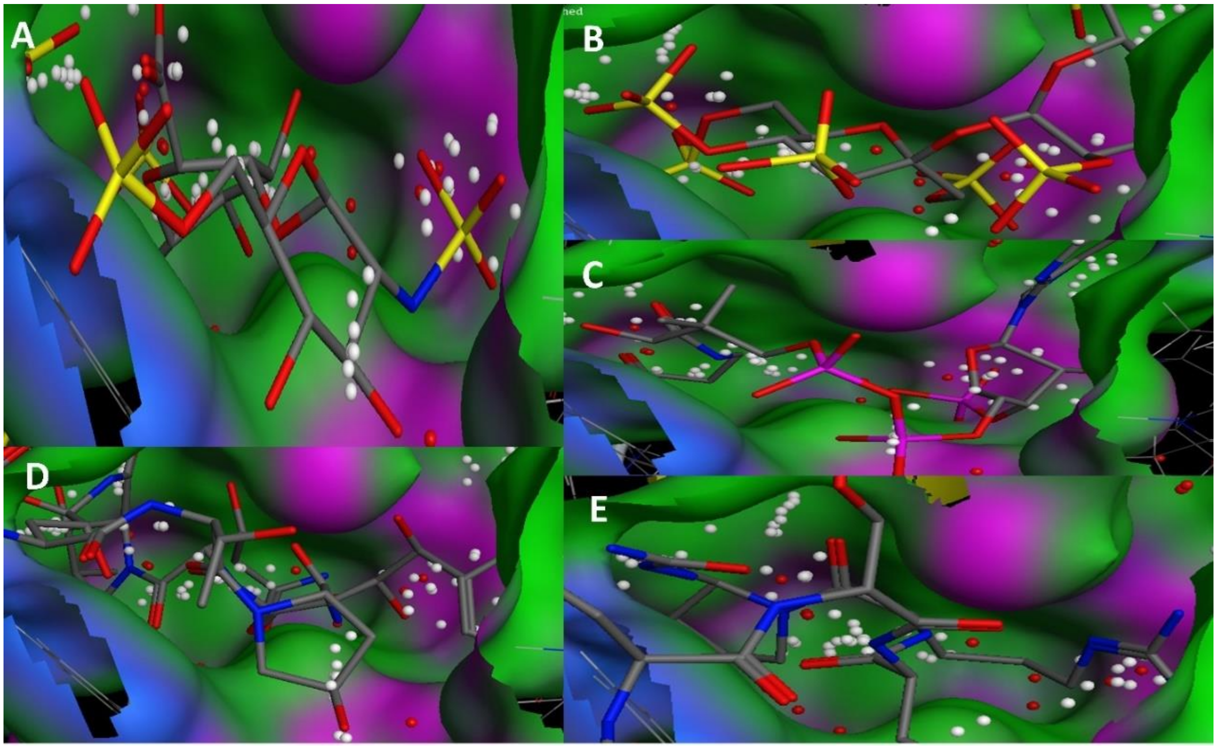

Figure (Supplementary F2): The docking binding mode of OMP25 protein and Drugbank database. (A) DB00569, (B) DB01901, (C) DB01141, (D) DB02516 and (E) DB00362

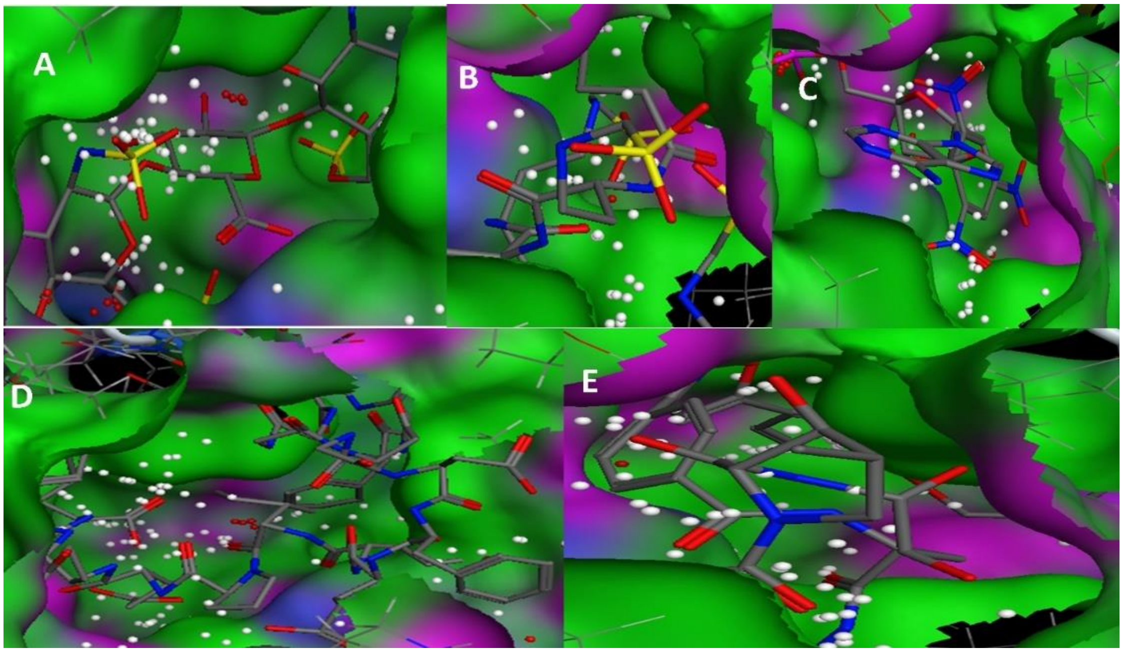

Figure (Supplementary F3): The docking binding mode of OMP31 protein & DrugBank database. (A) DB00569, (B) DB01111, (C) DB02524, (D) DB00006 and (E) DB01141
